# PTEN-negative endometrial cancer cells protect their genome through enhanced DDB2 expression associated with augmented nucleotide excision repair

**DOI:** 10.1186/s12885-023-10892-5

**Published:** 2023-05-04

**Authors:** Fathima Hameed J S, Anjali Devarajan, Devu Priya M S, Ahel Bhattacharyya, Mayur Balkrishna Shirude, Debasree Dutta, Parimal Karmakar, Ananda Mukherjee

**Affiliations:** 1 Rajiv Gandhi Centre for Biotechnology, Cancer Research Program, Thycaud, Poojappura, Thiruvananthapuram, Kerala 695014 India; 2 Manipal Academy of Higher Education, Manipal, Karnataka 576104 India; 3 Rajiv Gandhi Centre for Biotechnology, Regenerative Biology Program, Thycaud, Poojappura, Thiruvananthapuram, Kerala 695014 India; 4 Department of Life Science and Biotechnology, Jadavpur University, 188, Raja S.C. Mullick Road, Kolkata, West Bengal 700 032 India

**Keywords:** Endometrial cancer, PTEN, DDB2, DNA damage response, Nucleotide excision repair, Unscheduled DNA synthesis, RNA polymerase II

## Abstract

**Background:**

Endometrial cancer (EC) arises from uterine endometrium tissue and is the most prevalent cancer of the female reproductive tract in developed countries. It has been predicted that the global prevalence of EC will increase in part because of its positive association with economic growth and lifestyle. The majority of EC presented with endometrioid histology and mutations in the tumor suppressor gene PTEN, resulting in its loss of function. PTEN negatively regulates the PI3K/Akt/mTOR axis of cell proliferation and thus serves as a tumorigenesis gatekeeper. Through its chromatin functions, PTEN is also implicated in genome maintenance procedures. However, our comprehension of how DNA repair occurs in the absence of PTEN function in EC is inadequate.

**Methods:**

We utilized The Cancer Genome Atlas (TCGA) data analysis to establish a correlation between PTEN and DNA damage response genes in EC, followed by a series of cellular and biochemical assays to elucidate a molecular mechanism utilizing the AN3CA cell line model for EC.

**Results:**

The TCGA analyses demonstrated an inverse correlation between the expression of the damage sensor protein of nucleotide excision repair (NER), DDB2, and PTEN in EC. The transcriptional activation of DDB2 is mediated by the recruitment of active RNA polymerase II to the DDB2 promoter in the PTEN-null EC cells, revealing a correlation between increased DDB2 expression and augmented NER activity in the absence of PTEN.

**Conclusion:**

Our study indicated a causal relationship between NER and EC that may be exploited in disease management.

**Supplementary Information:**

The online version contains supplementary material available at 10.1186/s12885-023-10892-5.

## Background

Endometrial cancer (EC) is the most common gynecological malignancy in the USA [1] and has an increasing incidence and mortality rate predicted globally [2]. It often arises from the inner lining of the uterine corpus, the endometrium [3]. Being an estrogen-responsive tissue, unopposed estrogen exposure to postmenopausal endometrium and the increasing amount of circulating estrogen from abdominal fat of obese women inflict EC. Excess body weight and a sedentary lifestyle impose a greater disease risk [4]. The most established treatment procedures for women presented with EC are surgery, radiation, hormonal therapy, and/or chemotherapy, depending on disease progression. Immunotherapy and targeted therapy also showed promising outcomes in managing disease in specific situations [5]. The 5-year survival rate of EC with localized tumors is 82% though it is significantly reduced for distant tumors [6]. In general, the survival rate for common cancers has improved in past decades, except for a few cancers, including EC, which begs attention for a better therapeutic strategy [1].

The Cancer Genome Atlas (TCGA) published comprehensive genomic and transcriptomic analyses of EC, identifying four molecular subgroups: *POLE* ultramutated, microsatellite instability hypermutated, copy-number low, and copy-number high [7]. Except for the copy-number high, all three subgroups attribute endometrioid phenotype and a high percentage of genetic alterations in the tumor suppressor gene *PTEN*. In contrast, the copy number high group is characterized by serous histology with a high frequency of *TP53* gene mutation. Thus the loss of expression of PTEN served as a validated biomarker for the endometrioid subtype- the most common form of EC [8]. In addition, *PTEN* alterations are associated with favorable survival outcomes for EC patients [9], indicating the PTEN pathway as a potential molecular candidate for the targeted therapy in managing the disease.

Canonical PTEN negatively regulates the PI3K/AKT/mTOR signaling pathway by dephosphorylating the upstream kinase PIP3 through lipid phosphatase activity. Therefore, loss of the PTEN function leads to overactivation of the PI3K/AKT pathway and stimulation of cell proliferation and tumorigenesis [10]. On the other hand, the noncanonical nuclear functions of PTEN in maintaining genome stability and chromatin organization suggest its role as caretaker of the genome [11, 12].

Targeting PI3K/AKT/mTOR signaling axis in PTEN-negative EC has limited success in preclinical [13] and clinical studies [14], as PI3K inhibitors are often attributed to drug-related cytotoxicity and feedback upregulation of compensatory mechanisms [15]. Alternatively, targeting either DNA repair function alone [16] or combining PI3K signaling [17] might strengthen personalized therapeutic strategies in EC patients with *PTEN*’s loss-of-function mutations. In a recent study, we demonstrated that the loss of nuclear PTEN in EC accumulates DNA damage and increases sensitivity to the PARP inhibitor, olaparib [18].

Since successful targeting of DNA repair functions in EC requires comprehending the complex interaction of *PTEN* mutations with DNA damage repair (DDR) processes, the current study aims to understand the DNA repair mechanism in PTEN-negative EC. Here we report a functional association of nucleotide excision repair (NER) in EC through noncanonical functions of PTEN.

NER removes many types of DNA lesions from genomic DNA, including UV-induced DNA photolesions. The tight wrapping of genomic DNA around histones impedes DNA repair proteins from accessing DNA lesions buried in nucleosomal DNA. Damage-specific DNA binding protein 2 (DDB2) and Xeroderma pigmentosum complementation group C (XPC) protein complexes detect DNA lesions in nucleosomal DNA in mammalian global genome nucleotide excision repair (GG-NER), which scans the entire genome for damage and initiates repair. DNA damage recognition is the initial critical step influencing the overall efficiency of DNA repair [19].

## Methods

### Analysis of public databases

TCGA uterine corpus endometrial cancer dataset (TCGA-UCEC) [7] was downloaded from cBioPortal (https://www.cbioportal.org/datasets), and differential mRNA expression was analyzed for a subset of DDR genes from a TCGA Pan-cancer study [20] in PTEN altered and unaltered UCEC samples. Cell lines in Endometrial Adenocarcinoma dataset from Cancer Cell Line Encyclopedia (CCLE) [21, 22] was analyzed for gene and protein expression in *DDB2* and PTEN, respectively, using the DepMap portal (https://depmap.org/portal/context/endometrial_adenocarcinoma). Kaplan-Meier analysis of the correlation between *DDB2* mRNA expression level and patient survival data for EC was analyzed using the Human Protein Atlas pathology portal [23] (https://www.proteinatlas.org/ENSG00000134574-DDB2/pathology/endometrial+cancer). The disease-free tissue-specific gene expression profile for *PTEN* and *DDB2* was analyzed using the GTEx [24] multi gene query function (https://www.gtexportal.org/home/multiGeneQueryPage).

### Plasmids and sub-cloning

The pcDNA3-HA-PTEN plasmid was purchased from Addgene (catalog no. 78776). The full-length cDNA of *PTEN* was subcloned into pEGFP-C1 (Clontech, catalog no. 6084-1) by digestion with *Kpnl HF* (New England Biolabs, catalog no. R3142S), followed by a ligation reaction using T4 DNA ligase (New England Biolabs, catalog no. M0202S) with the vector pEGFP-C1 digested with the same restriction enzyme. An alkaline phosphatase treatment was performed using a Quick CIP kit (New England Biolabs, catalog no. M0525S) just before the ligation reaction to prevent re-legation of vector and insert. The orientation of the *PTEN* cDNA insert into the pEGFP-C1 was checked by restriction digestion with *Nhel* (New England Biolabs, catalog no. R0131), and the clones were confirmed by Sanger sequencing.

### Cell culture, transfection, and establishment of stable cell lines

The EC AN3CA cell lines were purchased from American Type Culture Collection (ATCC, catalog no. HTB-111). Cells were cultured in Dulbecco’s modified Eagle’s medium (DMEM) (Himedia, catalog no. 11330032) supplemented with 10% fetal bovine serum (FBS) (Thermo Fisher Scientific, catalog no. 10270106) and 1% antibiotic-antimycotic (Thermo Fisher Scientific, catalog no. 15240062) at 37 °C in a humidified environment containing 5% CO_2_. MCF 10A cell line was cultured in DMEM/F-12 (Gibco, catalog no. 11330032) supplemented with 5% horse serum ( Gibco, catalog no. 26050088), 20 ng/ml hEGF (Thermo Fisher Scientfic, catalog no. PHG0311), 0.5 µg/ml Hydrocortisone (Sigma-Aldrich, catalog no. H0888), 100 ng/ml cholera toxin (Sigma-Aldrich catalog no, C8052), 10 µg/ml insulin and 1% antibiotic-antimycotic (Thermo Fisher Scientific, catalog no. 15,240,062) at 37 °C in a humidified environment containing 5% CO_2_.

AN3CA cells grown to 60% confluency in tissue culture dishes were transfected with either empty vector pEGFP-C1 or pEGFP-C1-PTEN-FL plasmids using the Lipofectamine 3000 (Thermo Fisher Scientific, catalog no. L3000001) transfection reagent as described by the manufacturer.

After transfection, the cells were serially diluted into a 96-well plate, so the last well contained only a single cell. The transfected clones were then selected by growing the cells in the growth medium containing antibiotic geneticin (Thermo Fisher Scientific, catalog no. 10131035). The 96-well plates were screened for GFP-expressing single-cell colonies. AN3CA cell lines stably expressing empty vector pEGFP-C1 (Vector) and pEGFP-C1-PTEN-FL (PTEN-FL) were derived from the colonies displaying the highest GFP expression levels under a fluorescence microscope.

### UV irradiation to live cells

Cells were irradiated with varying UV doses (J/m^2^/sec) using a UV Crosslinker (VWR) equipped with five overhead 8 W UV lamps producing 254 nm wavelength (UVC).

### Determination of LD50 and cell death analysis

Vector and PTEN-FL cells were plated on a 96-well plate at a density of 1 × 10^4^ cells/well. The cells were then irradiated with different UV doses (0 J/m^2^, 2.5 J/m^2^, 5 J/m^2^, 10 J/m^2^, 25 J/m^2^, 50 J/m^2^, 100 J/m^2^, 250 J/m^2^, 500 J/m^2^, and 1000 J/m^2^), followed by further incubation at 37 °C for 4 h. 10 µl of MTT solution (5 mg/ml) (Sigma-Aldrich, catalog no. M5655) was added to 100 µl of media in each well incubated for 3 h at 37 °C. The purplish formazan crystals were dissolved in the dark with 100 µl of solubilizing buffer, DMSO (Sigma-Aldrich, catalog no. 41639). After the crystals completely dissolved, plates were swirled gently to make a uniform color, and absorbance was measured at 570 nm using a microtiter plate reader (Thermo Fisher Scientific). LD50 values were calculated by fitting the data into an inhibitory dose-response curve equation in GraphPad Prism software.

For analyzing the apoptosis, Vector and PTEN-FL cells seeded on 6-well plates were irradiated with a UV dose of 5 J/m^2^. After 4 h post-irradiation at 37 °C, cells were trypsinized (Thermo Fisher Scientific, catalog no. 25200072) and resuspended in 1 ml ice-cold PBS (Thermo Fisher Scientific, catalog no. 14190235). Annexin binding buffer and annexin V-APC (Thermo Fisher Scientific, catalog no. R37176) were then added to the cell pellets and incubated for 15 min at dark. 5 µl of propidium iodide (PI) (1 mg/ml) (Sigma-Aldrich, catalog no. P4170) was then added and incubated for 5 min at dark. The percentages of apoptotic cells were analyzed by flow cytometry.

### Flow cytometric analyses of cell cycle and unscheduled DNA synthesis (UDS)

The NER activity was measured by the amount of repair synthesis detected after UVC-induced damage [25] in the Vector and PTEN-FL cell lines. Cells were irradiated with a UVC dose of 5 J/m^2^. Immediately after UV exposure, cells were incubated with 5 µM click-chemistry compatible EdU (Thermo Fisher Scientific, catalog no. A10044) for 4 h in serum-free media. Cells were then fixed with fix buffer A (300 mM sucrose, 2% formaldehyde, 0.5% triton X-100), and blocked with 10% FBS in PBS, followed by azide coupling reaction for 1 h at dark to conjugate fluorophore Alexa Fluor 647 (Invitrogen, catalog no. A10277). After the reaction, cells were stained with PI in the dark for 20 min at room temperature. Flow cytometry analyses were performed to determine the UDS-positive cells in different cell cycle phases.

### Subcellular fractionation and western blot analysis

Subcellular fractionation was performed as described previously [26]. Vector and PTEN-FL cells grown to 80% confluency were trypsinized and washed with 1 ml PBS. 250 µl from the cell suspension was centrifuged and the pellets were lysed with 2% SDS-lysis buffer (2% SDS, 50 mM Tris-HCl (pH 7.4), 10 mM EDTA) followed by rigorous resuspension. This part of cell suspension was taken as whole-cell extract. The remaining cell suspension were centrifuged and lysed with cytoskeletal (CSK) buffer (300 mM sucrose, 100 mM NaCl, 3 mM MgCl_2_, 0.5% Triton-X-100, 1 mM EGTA, 10 mM PIPES) added with protease inhibitor (Roche, catalog no. 04693124001). The cell suspension was centrifuged and the supernatant was taken as soluble part of the cell, and the pellet was again lysed with 2% SDS-lysis buffer, and taken as the chromatin part of the cell suspension. The whole cell lysate and chromatin samples were then boiled and sonicated, followed by quantification and western blotting of the protein samples.

For western blotting, Vector, PTEN-FL and MCF 10A cells were lysed using RIPA buffer, and the total protein was extracted. BCA protein assay kit (Thermo Fisher Scientific, catalog no. 23227) was used to detect total protein concentration. 10 to 25 µg of total protein was loaded for sodium dodecyl sulfate-polyacrylamide gel electrophoresis (SDS-PAGE) and transferred to polyvinylidene fluoride (PVDF) membranes. The membranes were cut after the transfer before probing with antibodies according to the molecular weights. PVDF membranes were blocked in 1X TBST solution containing 5% non-fat dried milk or 5% BSA for 1 h at room temperature and incubated with diluted primary antibodies overnight at 4 °C. 1X TBST solutions were used to wash the membranes three times, 5 min each. PVDF membranes were then incubated with secondary antibodies raised against mouse (Jackson ImmunoResearch Laboratories, catalog no. 115-035-062) or rabbit (Jackson ImmunoResearch Laboratories, catalog no. 111-035-045) conjugated with horseradish peroxidase (HRP) for 1 h at room temperature. After washing the membranes three times with 1X TBST, the chemiluminescence detection was carried out using Clarity Max Western ECL Substrate (Bio-Rad, catalog no. 1705062). Using the ImageJ gel analysis tool, the band intensities were measured and values were represented as adjusted densities relative to their respective loading controls.

The primary antibodies used for the analyses were anti-GFP (Cell Signaling Technology, catalog no. 2956), anti-PTEN (Cell Signaling Technology, catalog no. 9188), anti-phospho-serine 473-Akt (Cell Signaling Technology, catalog no. 4051), anti-Akt (Cell Signaling Technology, catalog no. 9272), anti-DDB2 (Novus Biologicals, catalog no. NBP275718), anti-DDB1 (Abcam, catalog no. AB109027), anti-XPC (Novus Biologicals, catalog no. NB100-477), anti-XPB (Novus Biologicals, catalog no. NB100-61059), anti-Caspase-3 (Cell Signaling Technology, catalog no. 9668), anti-β-actin (Sigma-Aldrich, catalog no. A5441), anti-GAPDH (Sigma-Aldrich, catalog no. G9545), anti-H3 (Cell Signaling Technology, catalog no. 4499).

### Immunocytochemistry

Cells seeded on sterile 18 mm coverslips in a 12-well plate were subjected to indirect co-immunofluorescence labelling at 50% confluency. The cells were fixed with the buffer containing 4% paraformaldehyde for 15 min and permeabilized with 0.5% of Triton X-100 for 10 min. The cells were then blocked with 5% BSA in PBST for 1 h at room temperature, followed by anti-GFP (Cell Signaling Technology, catalog no. 2956) and anti-PTEN antibody (Cell Signaling Technology, catalog no. 9188) incubation for overnight at 4 °C. After multiple washing, the cells were labelled with Alexa Fluor 488 (Invitrogen, catalog no. A11034) and 633 (Invitrogen, catalog no. A21052) conjugated secondary antibodies for 1 h at room temperature, followed by DAPI staining of the nuclei (Sigma-Aldrich, catalog no. D9542) for 15 min at room temperature in dark. Coverslips were mounted onto glass slides, and confocal microscopy was performed for image acquisition. Image analyses were conducted using ImageJ software.

### RNA isolation and RT-qPCR analysis

Total RNA was extracted from the cells using TRIzol reagent (Invitrogen, catalog no. 15596026). The concentration and purity of total RNA were measured using NanoDrop (Thermo Fisher Scientific). Reverse transcription reactions were performed using the High-Capacity cDNA Reverse Transcription Kit (Thermo Fisher Scientific, catalog no. 4368814). The cDNA was amplified by gene specific primers using the SYBR Green PCR Master Mix (Thermo Fisher Scientific, catalog no. 4367659) for RT-qPCR in the StepOnePlus real-time PCR system (Applied Biosystems). Primers for *DDB2* gene were:

Forward: 5’ GAGCTTCCTGGCCATCTGT 3’.

Reverse: 5’ GGGCAGCCTTTTGTAATATCC 3’.

Primers for *DDB1* gene were:

Forward: 5’ ATTGCGGTCATGGAGCTTT 3’.

Reverse: 5’ CAGGATGCAGGCATTGTACTT 3’.

Primers for housekeeping gene *GAPDH* were:

Forward: 5’ CACCAGGGCTGCTTTTAACTCTGGTA 3’.

Reverse: 5’ CCTTGACGGTGCCATGGAATTTGC 3’.

### Chromatin immunoprecipitation

The cells were subjected to ChIP analysis as previously described [27]. Briefly, AN3CA cells were irradiated with 5 J/m^2^ of UV dose. After trypsinization, 1 × 10^6^ cells per assay were crosslinked with methanol free 1% formaldehyde (Thermo Fisher Scientific, catalog no. 28908) and sonicated to generate chromatin fragments. Protein-DNA crosslinks were precipitated using antibody against RNA polymerase II carboxy-terminal domain phospho-serine 5 (Active Motif, catalog no. 39233). Primers were designed to measure the enrichment of chromatin fragments by RT-qPCR. SYBR Green reporter intensity was measured relative to the standard curve of input chromatin prepared from Vector and PTEN-FL cells. IgG (Vector Laboratories, catalog no. I-1000-5) was used as the negative control. Primers for *DDB2* promoter were:

Forward: 5’ TGAGCGACAGAGCCAGACC 3’.

Reverse: 5’ CCGAGCTAAGCCAACTTCC 3’.

### Statistical analysis

Quantitative data were described as mean ± standard deviation (SD) values of the mean or median values with 95% confidence interval (CI). Representative data from three independent experiments were shown for immunocytochemistry, western blot, and flow cytometry images. Two-group comparisons were conducted by the paired Student’s *t*-test. Multi-group comparisons were conducted by two-way ANOVA and Tukey’s multiple comparison tests. The specific statistical tests applied to the experiments were mentioned in the respective figure legend. Statistical analyses were performed using GraphPad Prism 9 (GraphPad Software).

## Results

### PTEN negatively correlates with ***DDB2*** expression in endometrial cancer

**Fig. 1:**
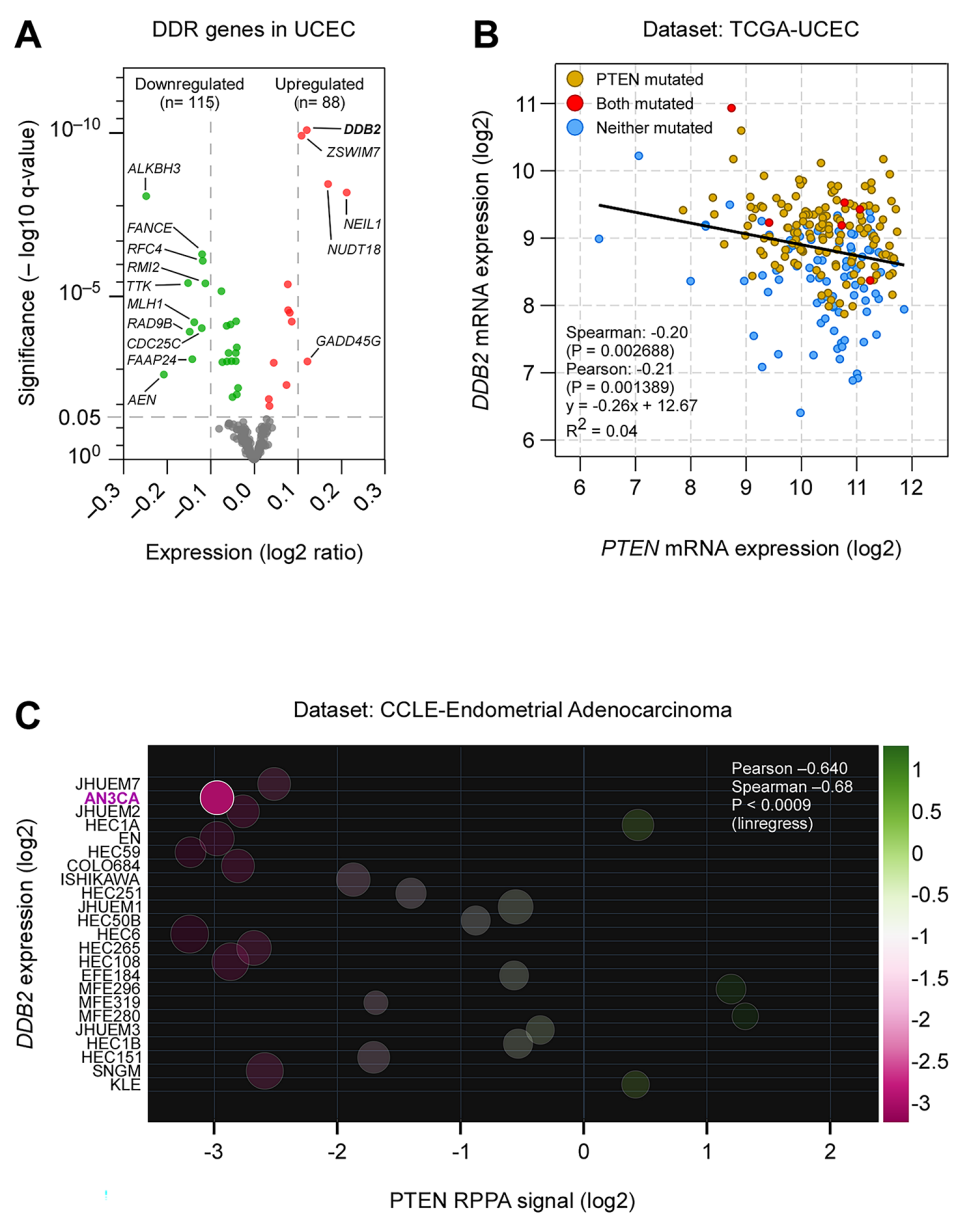
DDR genes in EC. (A) Analysis of differential expression of DDR genes based on *PTEN* genetic alteration in TCGA-UCEC [7]. The list of DDR genes curated elsewhere [20] was previously obtained from the PAN-cancer TCGA study. The volcano plot presented the statistical significance and magnitude of change of DDR genes as a ratio of PTEN-altered expression values to unaltered expression values. The green, red, and gray dots, respectively, represent genes that were significantly downregulated, significantly upregulated, and not significant. (B) The scatter plot depicted a correlation between *PTEN* and *DDB2* mRNA expression in UCEC. The correlation plot was generated by the cBioPortal algorithm. (C) The scatter plot illustrated a correlation between PTEN protein expression and *DDB2* mRNA expression in 23 endometrial cancer cell lines. Data were obtained from CCLE in DepMap portal and analyzed using Plotly Chart Studio. The variable color scale represented the reverse-phase protein array (RPPA) signal of PTEN, and the area of each circle represented *DDB2* mRNA expression in the individual cell lines. All statistical analyses of the datasets were obtained directly from the respective portals

Being an ultrahypermutant group of tumors [28], EC harbors the highest mutation load in DDR genes [20]. Therefore, we first examined the expression of 276 DDR genes in EC, curated by Knijnenburg et al., across 33 cancer types from TCGA analysis [20]. We found 203 among 276 DDR genes associated with uterine corpus endometrial carcinoma (UCEC) dataset [7] in TCGA (TCGA-UCEC) (Table S1). We calculated the log fold change for DDR genes as the ratio of PTEN-altered to unaltered expression values from the same TCGA-UCEC dataset and plotted against their statistical significance corrected for multiple comparisons to investigate their differential expression (Fig. 1A). We observed that damage specific DNA binding protein 2 (*DDB2*) is the most significantly upregulated gene in this cohort. *DDB2* gene encodes a protein crucial for sensing photolesions in DNA induced by ultraviolet (UV) radiation [29]. This gene mutation is responsible for Xeroderma pigmentosum complementation group E (XPE), a phenotype sensitive to UV light due to defective NER [30]. Importantly, we observed a significant negative correlation of mRNA expressions between *PTEN* and *DDB2* in the same TCGA-UCEC dataset (Fig. 1B). However, normal uterine tissues display similar transcript levels of *PTEN* and *DDB2* (Fig. S1A) in the Genotype-Tissue Expression (GTEx) database portal [24]. Clinically, the high expression of *DDB2* is prognostic and associated with favorable survival outcomes in EC patients [23] (Fig. S1B).

Next, we checked the Cancer Cell Line Encyclopedia (CCLE), an open resource of large-scale genetic and chemical characterizations of cancer cell lines [21, 22], in a search for an EC cell line to validate the correlation of the expressions between *PTEN* and *DDB2* observed in TCGA. Among 23 EC cell lines, we noticed that AN3CA has one of the highest expressions of the *DDB2* gene in the absence of PTEN (Fig. 1C). The PTEN protein is non-functional in the AN3CA cell line due to a frame-shift deletion, but there is no genetic alteration in the *DDB2* gene. Therefore, we procured an AN3CA cell line from ATCC to test the hypothesis whether the enhanced expression of *DDB2* in PTEN-negative EC is associated with augmented NER.

**Fig. 2:**
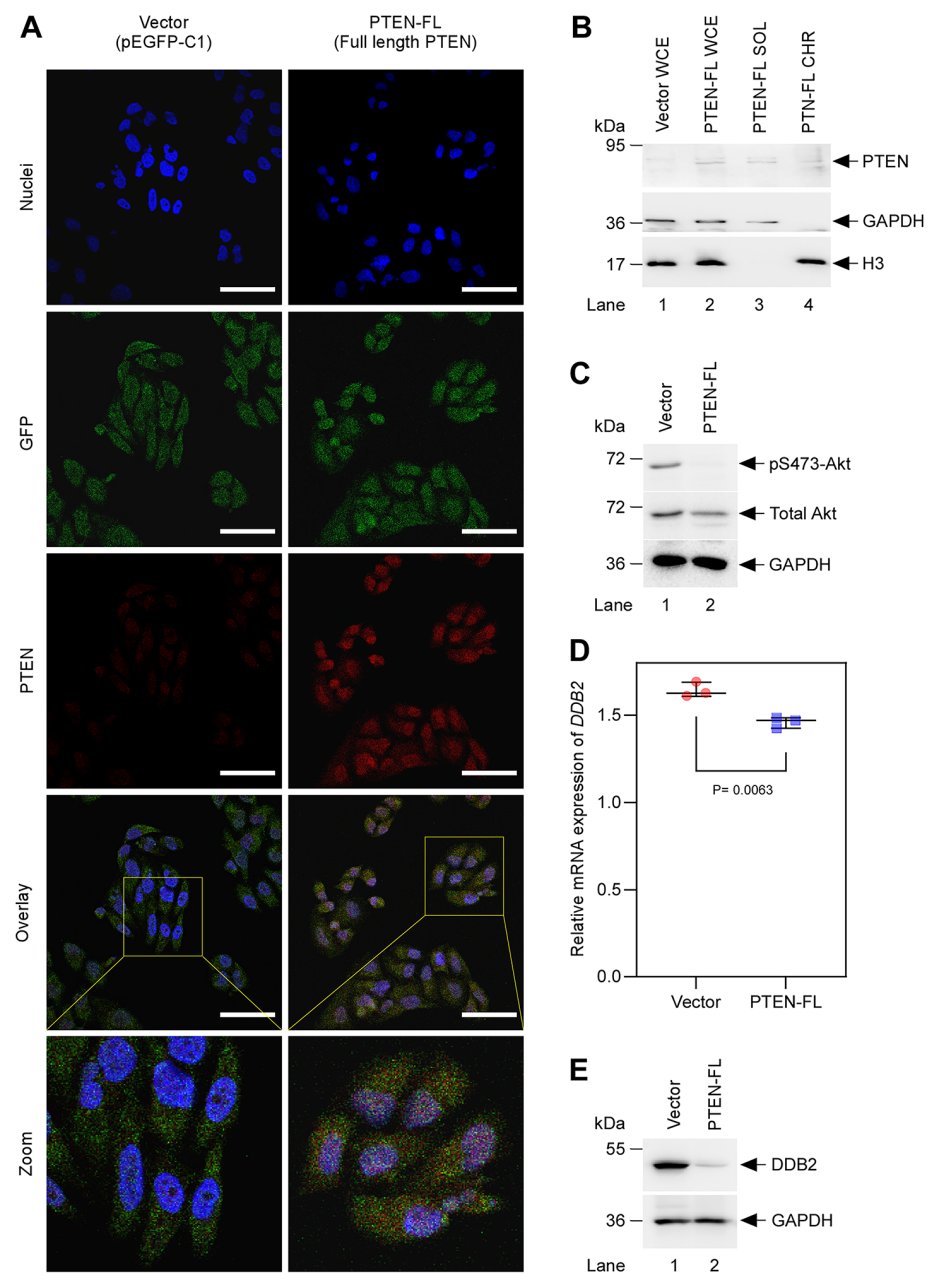
Reconstitution of PTEN in PTEN-null AN3CA EC cell line. (A) Immunofluorescent detection of PTEN (red) after stable expression of full-length *PTEN* cDNA in AN3CA, labeled as PTEN-FL. AN3CA cells stably expressing empty pEGFP-C1 control plasmid (green) labeled as Vector. In the overlay panels, yellow squares indicated the areas that were magnified in the zoom panels. Scale bar 50 μm. (B) Immunoblot detected PTEN in PTEN-FL cells after cell fractionation. WCE, SOL, and CHR represented whole-cell extract, soluble, and chromatin fractions, respectively. GAPDH and H3 were used as loading control for soluble and chromatin fractions, respectively. (C) Immunoblot detected phosphorylated Akt at serine 473 residues and total Akt in the whole-cell extracts. GAPDH was used as a loading control. (D) Relative expression of the *DDB2* gene was determined by RT-qPCR. The expression values were normalized to an internal control *GAPDH*. A paired t-test was applied to three independent biological replicates for statistical comparison. (E) Immunoblot detected DDB2 in the whole-cell extracts. GAPDH was used as a loading control

We sub-cloned the full-length cDNA of PTEN from pcDNA3-HA-PTEN into a pEGFP-C1 background and to validate, we used anti-GFP (Fig. S2A) and anti-PTEN (Fig. S2B) antibodies to detect GFP and fusion PTEN- GFP bands after overexpression of pEGFP-C1 and pEGFP-C1-PTEN-FL plasmids in AN3CA cells, respectively. A non-tumorigenic breast epithelial cell line, MCF 10A whole-cell lysate, was utilized as a negative control for GFP expression and a positive control for endogenous PTEN expression. Next, we established either a stably transfected clone of AN3CA cell line from a single cell expressing full-length PTEN-GFP fusion protein (henceforth called PTEN-FL) or GFP only for the empty vector (henceforth called Vector). Using these syngeneic AN3CA cell lines, we checked the expression of PTEN and GFP by indirect co-immunolabeling with anti-PTEN and anti-GFP antibodies. As anticipated, PTEN-FL cells expressed PTEN, but Vector cells did not, whereas reporter GFP was expressed in both Vector and PTEN-FL cell lines (Fig. 2A). Moreover, we observed that the fusion PTEN-GFP protein was expressed throughout the cytoplasm and the nuclei of the PTEN-FL cells. To validate it biochemically, one step further, we checked the soluble and chromatin fractions from PTEN-FL cells along with whole-cell extracts (WCE) from both Vector and PTEN-FL cells for PTEN expression. PTEN was expressed in the WCE, soluble and chromatin fractions of PTEN-FL cells, whereas there was no expression of PTEN in the WCE of Vector cells (Fig. 2B). Although we observed a low level of PTEN expression in the PTEN-FL WCE, we detected a faint PTEN band in the chromatin fraction. This observation is in-line with our previous report [18] and reports from the other groups [11, 31], which describe its nuclear functions in maintaining genomic integrity. PTEN regulates the PI3K/Akt/mTOR pathway by dephosphorylating PIP3 to PIP2, thus limiting the downstream Akt phosphorylation [32]. We confirmed this by checking the phosphorylation level of serine 473 residues of Akt (pS473-Akt) after the reconstitution of PTEN. We did not detect any expression of pS473-Akt in PTEN-FL cells compared to the Vector cells (Fig. 2C). Together these results suggest that we successfully reconstituted a full-length PTEN in the PTEN-null AN3CA cell line with both chromatin and cytoplasmic functions intact.

We analyzed the relative mRNA expression of *DDB2* in Vector and PTEN-FL cells to validate its correlation with PTEN that we observed earlier in the TCGA -UCEC dataset [7]. Indeed, we found that the reconstitution of PTEN significantly reduced the mRNA expression of *DDB2* compared to Vector cells (Fig. 2D). The same hold at the protein level as we performed the western blot analysis probing with an anti-DDB2 antibody (Fig. 2E). Interestingly, the expression of DDB1, the binding partner of DDB2 in the UV-DDB complex, XPC, the key protein responsible for damage recognition in GG-NER, and XPB, a subunit of TFIIH that unwinds the DNA at the damage site, was not significantly altered in these cells (Fig. S3). This critical piece of data, along with the TCGA-UCEC dataset analyses, advises a negative correlation of loss of function of PTEN with DDB2 expression and thereby suggests an implication of NER in EC.

### Higher expression of DDB2 associates with augmented NER in endometrial cancer

**Fig. 3:**
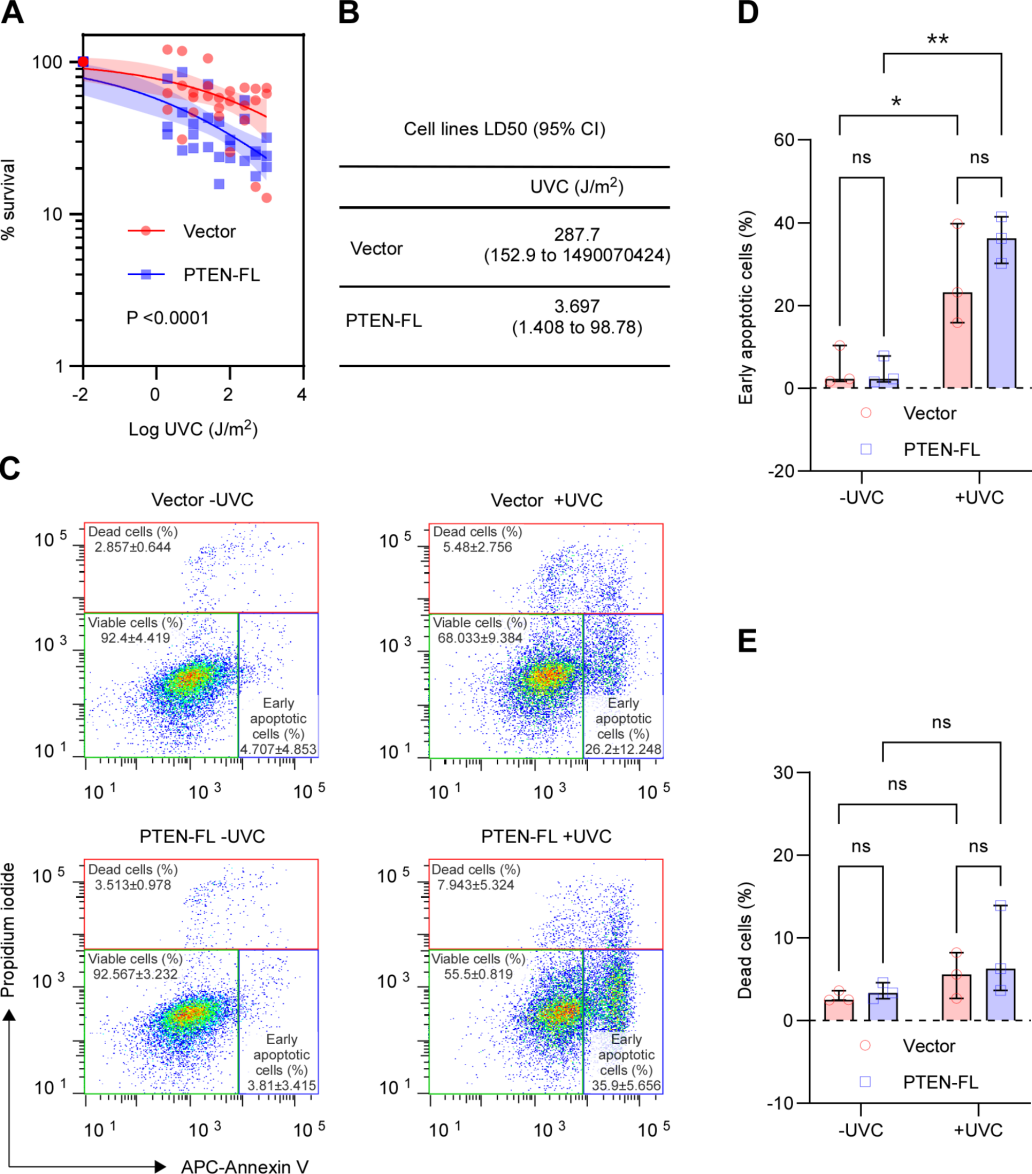
Effects of UV light-induced DNA damage on cell viability in EC. (A) A survival plot for Vector and PTEN-FL cells. Cell survivals were determined at 4 h post-irradiation with UVC (254 nm) light by MTT assay. The doses of UVC were transformed into a log scale. A variable slope least square fit was applied to normalized responses of three independent biological replicates to compare fits. The shaded areas represented the error band of 95% CI. (B) The table described the LD50 values of Vector and PTEN-FL cells with 95% CI. (C) Flow cytometric analyses of cell deaths. Cell deaths were determined at 4 h post-irradiation with a 5 J/m^2^ UVC dose by staining the cells with annexin V conjugated with fluorophore APC and labeling the DNA with PI. The values in the representative scatter plots were expressed as mean and standard deviation. (D) The plot described the percentages of early apoptotic cells of three independent biological replicates as median and 95% CI. A two-way ANOVA test was performed to compare multiple groups. (E) The plot described the percentages of dead cells of three independent biological replicates as median and 95% CI. A two-way ANOVA test was performed to compare multiple groups. * P < 0.05, ** P < 0.01, ns, not significant

UV radiation to cells induces a broad spectrum of genomic lesions destabilizing the DNA backbone. After being recognized by UV-DDB damage sensing complex, the distortions in DNA helix are eliminated by the GG-NER [19]. Therefore, we irradiated the Vector and PTEN-FL cells with varying doses of UV light of 254 nm wave length (UVC) (Fig. 3A) and determined the LD50 values for both cell lines at four hours post-irradiation. We noticed a significant 77.81 fold higher LD50 value for the Vector cell (287.7 J/m^2^) compared to the PTEN-FL cells (3.697 J/m^2^) (Fig. 3B), suggesting a greater extent of UVC sensitivity in PTEN-FL cells. Therefore, we opted for a sub-lethal 5 J/m^2^ UVC dose for cell irradiation and performed the PI and annexin V double staining assay at four hours post-irradiation to measure the early apoptotic and dead cells using flow cytometry (Fig. 3C). Despite the increase in the percentage of early apoptotic cells after UVC irradiation in both Vector and PTEN-FL cells, there was no significant difference between Vector and PTEN-FL cells (Fig. 3D). Likewise, we found no significant effects of UVC irradiation in dead cell percentages between Vector and PTEN-FL cells (Fig. 3E). Using anti-Caspase-3 antibody, an additional western blot experiment confirmed the results of the flow cytometric analysis of apoptosis (Fig. S4.). Hence, we used a 5 J/m^2^ UVC dose to activate and functionally measure the GG-NER activity in the cells.

**Fig. 4:**
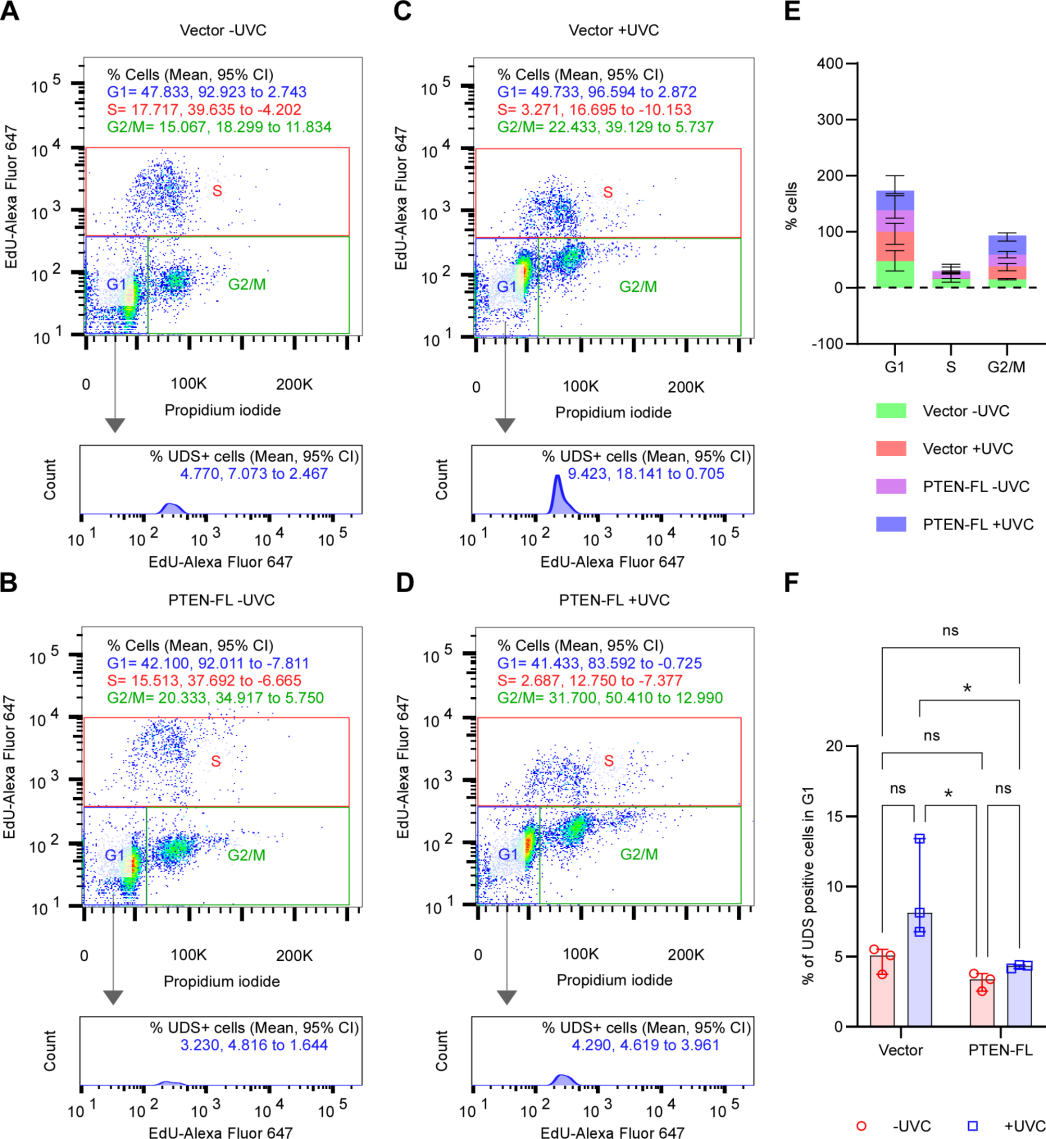
Effects of UV light-induced DNA damage on cell cycle and NER activity in EC. A-D. Flow cytometric analyses of cell cycle and unscheduled DNA synthesis (UDS) of either un-irradiated Vector (A) and PTEN-FL cells (B) or 5 J/m^2^ UVC irradiated Vector (C) and PTEN-FL (D) at 4 h post-irradiation. Cells were analyzed after the incorporation of EdU, followed by AlexaFluor 647 conjugation and DNA staining with PI for the cell cycle (upper panels) and the UDS at the G1 phase (lower panels). The values in the representative scatter plots (upper panel) and histograms (lower panels) were expressed as mean and 95% CI. E. The stacked bar plot described the percentages of G1, S, and G2/M phases cells of three independent biological replicates as median and 95% CI. A two-way ANOVA test was performed to compare multiple groups. F. The plot described the percentages of UDS-positive cells in G1 phase of three independent biological replicates as median and 95% CI. A two-way ANOVA test was performed to compare multiple groups. * P < 0.05, ns, not significant

We have utilized flow cytometric detection of non-radioisotopic labeling of nucleotide precursors incorporated into DNA during synthesis. The method measures the repair synthesis step in NER that occurs outside the S-phase [33], hence called unscheduled DNA synthesis (UDS), which could be a read-out for NER activity [25]. We pulse-labeled the cells with 5-ethynyl-2’-deoxyuridine (EdU) immediately after UVC treatment and measured the percentages of the cells in G1, S, and G2/M phases of cell cycles at four hours post-irradiation (Fig. 4A-D, upper panels). Despite a reduction in the percentage of S-phase cells after UVC irradiation, we found no significant difference among the different treatment groups of the cells (Fig. 4E). From the same experiments, we determined the number of EdU-positive cells in G1 (Fig. 4A-D, lower panels) and G2/M (Fig. S5A) phases and quantified the percentages of these cells, representing UDS after UVC treatment. Interestingly, in the G1 population, we observed a significantly higher percentage of UDS-positive cells in Vector than PTEN-FL after UVC treatment (Fig. 4F). However, despite an apparent increase in the UDS-positive cell population in the G2/M post-UVC Vector group (Fig. S5A), we did not observe any statistically significant difference between Vector and PTEN-FL cells (Fig. S5B). This data and initial observations suggest that higher expression of DDB2 could be the function of increased NER activity in the PTEN-negative EC cells.

### Transactivation of ***DDB2*** is mediated by RNA polymerase II through the chromatin function of PTEN in endometrial cancer

**Fig. 5:**
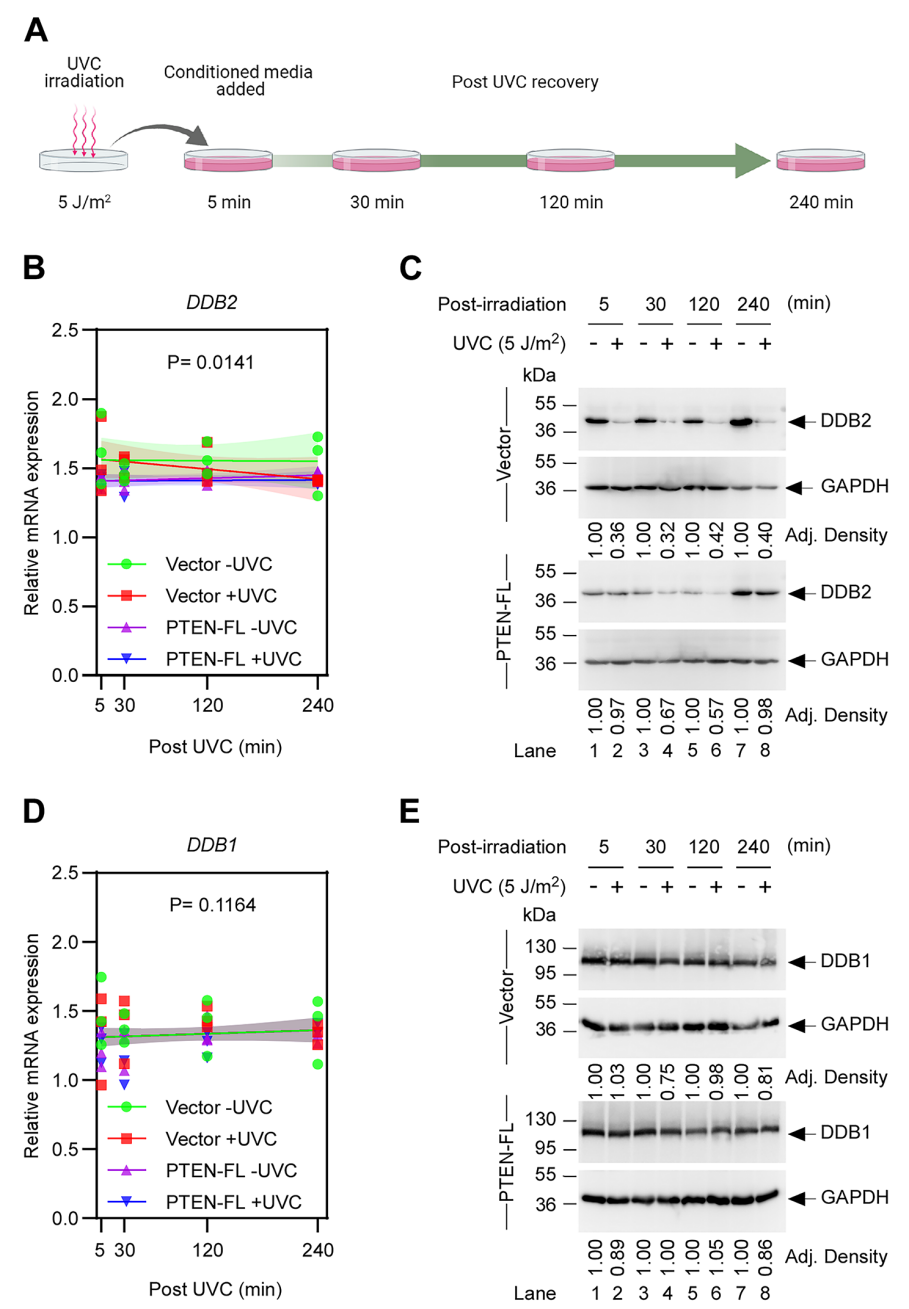
Temporal expression kinetics of UV-DDB in Vector and PTEN-FL cells. (A) Diagram illustrating the timeline of samples collection in post-UVC-irradiation. (B) Relative mRNA expression of the *DDB2* was determined by RT-qPCR at the indicated post-irradiated (5 J/m^2^) time points. The expression values were normalized to an internal control *GAPDH*. A centered first order polynomial least squares fit was applied to three independent biological replicates to compare fits. The shaded areas represented the error band of 95% CI. (C) Immunoblot detected DDB2 at the indicated time points in the whole-cell extracts. GAPDH was used as a loading control. The band intensities were measured as adjusted density (Adj. Density) relative to GAPDH. (D) Relative mRNA expression of the *DDB1* was determined by RT-qPCR at the indicated post-irradiated (5 J/m^2^) time points. The expression values were normalized to an internal control *GAPDH*. A centered first order polynomial least squares fit was applied to three independent biological replicates to compare fits. The shaded areas represented the error band of 95% CI. (E) Immunoblot detected DDB1 at the indicated time points in the whole-cell extracts. GAPDH was used as a loading control. The band intensities were measured as adjusted density (Adj. Density) relative to GAPDH.

Next, we were interested in checking the temporal kinetics of *DDB2* mRNA expression upon activation of NER in the absence and the presence of PTEN as it is negatively correlated with *DDB2* transcripts. To test this, we collected samples at four different time points from UVC post-irradiated cells (Fig. 5A). When we analyzed the expression of *DDB2* mRNA, linear regression confirmed a significant difference among the different treatment groups (Fig. 5B). Moreover, in contrast to the Vector cells, we observed longer retention of DDB2 protein in the PTEN-FL cells after UVC treatment from the corresponding western blot experiment (Fig. 5C), suggesting a slower ubiquitination-mediated proteasomal degradation impeding the next step of the repair process through Xeroderma pigmentosum complementation group C (XPC) damage recognition complex [34]. Furthermore, we tested whether DDB1, a component of the GG-NER damage sensor and a direct binding partner of DDB2 [29], has similar expression kinetics. Nonetheless, when we employed the same analyses, we observed no significant differences in either mRNA (Fig. 5D) or protein (Fig. 5E) expression levels of DDB1. This result indicates that the higher expression of DDB2 in the absence of PTEN in EC cells is a distinctive phenomenon.

**Fig. 6:**
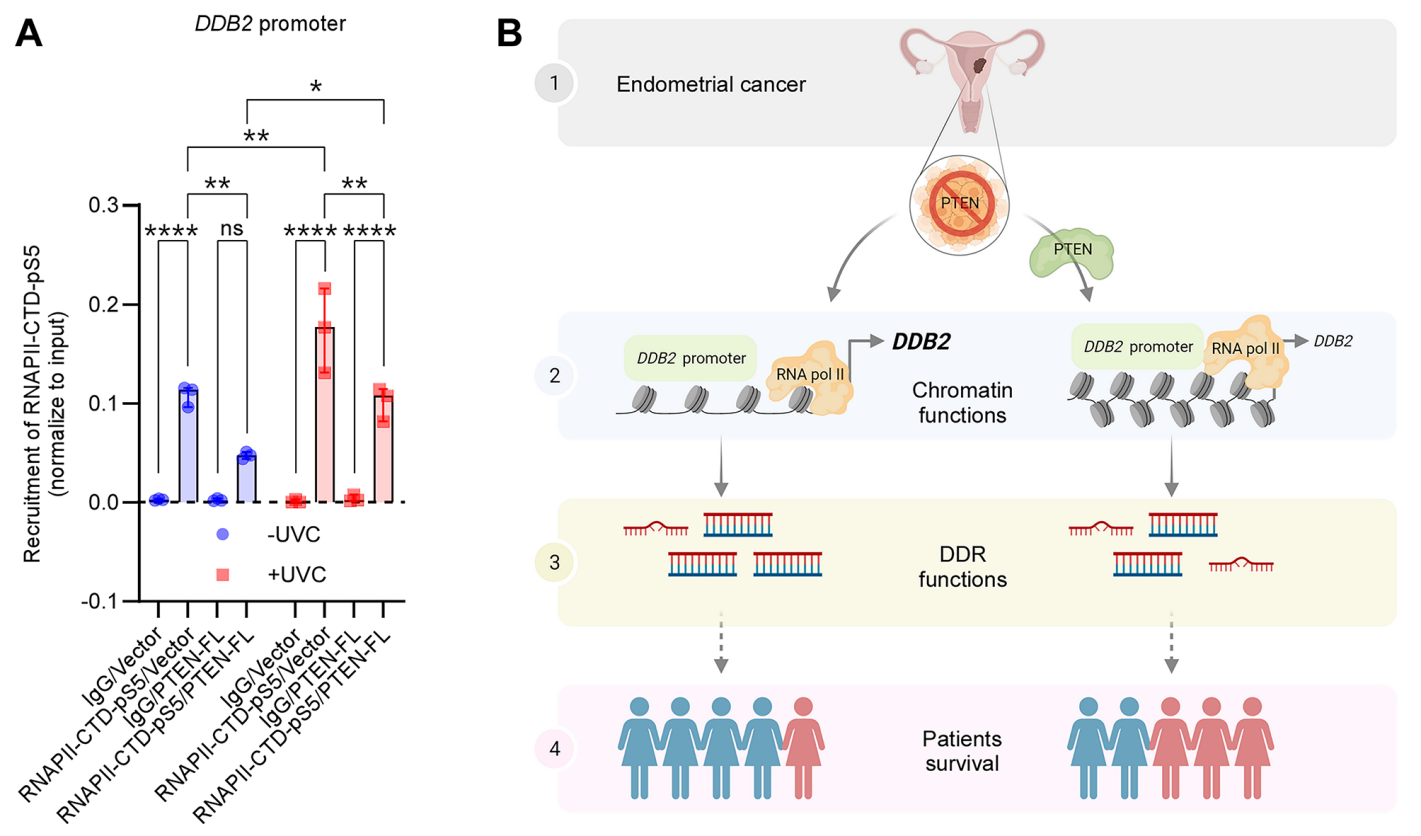
RNA polymerase II in *DDB2* transactivation. (A) The plot described the recruitment of phosphorylated serine 5 residue of RNAPII-CTD on *DDB2* gene promoter in unirradiated and irradiated (UVC 5 J/m^2^) Vector and PTEN-FL cells at four hours post-irradiation by ChIP assay. Values were normalized to input and expressed as median and 95% CI of three independent biological replicates. A two-way ANOVA test was performed to compare multiple groups. * P < 0.05, ** P < 0.01, **** P < 0.0001, ns, not significant. (B) A model depicted a probable mechanism and consequence of high DDB2 expression in PTEN-null EC. Women in blue represented live patients; women in red represented deceased patients

Based on the current understanding, the global transcription slows down in response to UV irradiation while the individual genes associated with DNA damage response are highly upregulated [35]. Whether the upregulation of DDB2 linked with increased NER activity in the absence of PTEN fits into this context, we checked if PTEN could control the transcription of *DDB2* as PTEN was recently implicated in transcription through regulating RNA polymerase II (RNAPII) [36–38]. The phosphorylation at the serine 5 (Ser5) residues of the carboxy-terminal repeat domain (CTD) of RNAPII marks the initiation of transcription [39]. Therefore, we performed the chromatin immunoprecipitation (ChIP) assay to check the recruitment of phosphorylated Ser5 CTD of RNAPII (phospho-Ser5 CTD RNAPII) at the *DDB2* promoter in the absence and the presence of PTEN after four hours post-UVC irradiation (Fig. 6A). Despite a substantial increase in phospho-Ser5 CTD RNAPII recruitment in both Vector and PTEN-FL cells after UVC irradiation, we observed a significant loss of the same in the presence of PTEN irrespective of the irradiation. This result suggests that loss in phospho-Ser5 CTD RNAPII recruitment could be a reason for the decreased expression of *DDB2* transcripts after the reconstitution of PTEN in PTEN-null AN3CA cells.

Combining our data, we postulate a model that suggests PTEN-negative EC induces the transactivation of *DDB2* by facilitating the recruitment of active RNA polymerase II at the *DDB2* promoter. Higher expression of DDB2 leads to augmented repair synthesis and protects the genome more efficiently, presumably contributing to the better survival of those patients (Fig. 6B).

## Discussion

In this study, we have presented a mechanistic insight into an observation that DDB2 and PTEN expression negatively correlates in EC. This phenomenon is not normal. GTEx data on the disease-free uterine tissues suggests very comparable expression patterns for *DDB2* and *PTEN* transcripts [24]. However, the expression pattern varies along the female reproductive tract due to the tissue specificity. Interestingly, irrespective of sunlight exposure, the normal skin tissues and cultured fibroblast cells show gene expression correlation patterns of *DDB2* and *PTEN* similar to EC (Fig. S1A). The possibility could be that the higher tissue-specific *DDB2* expression is also associated with cell survival functions. Indeed, emerging evidence suggests that DDB2 could regulate chromatin states, gene transcription, cell cycle progression, and protein degradation [40]. However, its current role in carcinogenesis is debatable as it could act as anti-oncogenic and pro-oncogenic in the context of cancer hallmarks [40, 41].

We showed that the lack of functional PTEN contributed to the transactivation of DDB2, which, in turn, protects the genome of EC cells through augmented NER activity. This phenomenon could partly be a reason for the favorable survival of EC patients attributed to high *DDB2* expression that we observed (Fig. S1B) in the Human Protein Atlas portal [42] for TCGA data [23]. Nonetheless, the inadequate evidence on endogenous DNA damages that trigger tissue-specific NER makes it challenging to establish a direct relation between NER and EC.

We observed that neither the absence nor the re-expression of PTEN impacted the UVC-induced apoptotic cell population in EC (Fig. 3D). Therefore, the significant difference in LD50 of UVC between Vector and PTEN-FL cells could be the ability of repair synthesis associated with DDB2 expression. The protective nature of DDB2 is not through apoptosis but augmented NER activity, particularly against UV-induced DNA damage, was elegantly demonstrated by Alekseev et al. [43].

We have not seen any significant G1 or G2/M cell cycle arrest in post-UVC-irradiated cells (Fig. 4E) as at the low dose of UVC (5 J/m^2^), which was the case here, the functional NER pathway could quickly and efficiently repair the damages without activating the G1 or the G2/M checkpoints [44]. Interestingly, we noticed that irrespective of the UVC-irradiation, the accumulation of UDS-positive cells was not significantly different in the G1 phase of both Vector and PTEN-FL cells (Fig. 4F), whereas it was markedly different in the G2/M phase of the cell cycle (Fig S2B). This discrepancy is presumably because the G1 chromatin is more easily accessible to NER machinery than relatively condensed G2/M chromatin [45]. Additionally, as we previously mentioned, NER responds faster to low UVC dose-induced damage [44], which might help bring down the percentages of UDS cells closer to baseline after four hours post-irradiation in the G1 cells.

DDB1, CUL4A/B, and RBX, as part of the UV-DDB, form an E3 ubiquitin ligase complex that ubiquitinates histone H2A after DDB2 recognizes the photolesion on the chromatin. This results in the dissociation of DDB2 from the chromatin via auto-polyubiquitination, thereby allowing the recruitment of XPC. In addition, DDB1-CUL4A/B-RBX stabilizes XPC on damaged chromatin through monoubiquitination [46–48]. Therefore, DDB2 must be rapidly degraded by proteasomes in order to transfer the repair process to XPC [34, 49]. Consequently, our observation of longer retention of DDB2 after UVC-damage might additionally contribute to impeding NER activity along with a lower DDB2 expression in the presence of PTEN. Moreover, our observation that DDB1 is unaffected by UV irradiation is well supported [46], as its recruitment at the photolesion site is dependent on DDB2 [50] and not its expression.

Although tumor suppressor p53 is implicated in the transcriptional activation of DDB2 [51], its involvement was found to be in the late stage of UV-induced damage when most of the NER activity receded [46]. In this study, we revealed the functional consequences of UVC-irradiation in the absence and presence of PTEN at the early stage of active NER.

PTEN, however, controls chromatin condensation by interacting with histone H1; thus, loss of PTEN induces the state of abnormal chromatin decondensation that leads to gene activation [52]. More direct evidence of PTEN-associated transactivation came from the recent studies that demonstrated the interaction of PTEN with the transcription machinery [37, 38] and regulation of genome-wide occupancy of RNA polymerase II in the context of chromatin by PTEN [36, 38].

## Conclusions

Together we established a model that depicts a causal relation between NER and EC through a noncanonical function of PTEN involving transcriptional regulation of the NER-associated gene, *DDB2*. This fundamental knowledge could be instrumental in developing a therapeutic strategy to target NER in EC.

## Electronic supplementary material

Below is the link to the electronic supplementary material.

Supplementary Material 1

Supplementary Material 2

## Data Availability

The datasets generated and/or analyzed during the current study are available in the TCGA repository [https://www.cbioportal.org/datasets], CCLE repository [https://depmap.org/portal/context/endometrial_adenocarcinoma], Human Protein Atlas pathology repository [https://www.proteinatlas.org/ENSG00000134574-DDB2/pathology/endometrial+cancer], and GTEx repository [https://www.gtexportal.org/home/multiGeneQueryPage].

## Abbreviations

Akt: Ak strain transforming
ATCC: American Type Culture Collection
CCLE: Cancer Cell Line Encyclopedia
CTD: C-terminal repeat domain
CRL4: CULLIN4 (CUL4) RING ligase
DAPI: 4′,6-diamidino-2-phenylindole
DDB2: Damage specific DNA binding protein 2
DDR: DNA damage repair
EC: Endometrial Cancer
EdU: 5-ethynyl-2’-deoxyuridine
GG-NER: Global Genome Nucleotide Excision Repair
GTEx: Genotype-Tissue Expression
mTOR: mammalian target of rapamycin
MTT: 3-(4,5-dimethylthiazol-2-yl)-2,5-diphenyltetrazolium bromide
NER: Nucleotide Excision Repair
PARP: Poly (ADP-ribose) polymerase
PI3K: phosphatidylinositol-3-kinase
PI: Propidium iodide
PIP2: Phosphatidylinositol 4,5-bisphosphate
PIP3: Phosphatidylinositol 3,4,5-trisphosphate
POLE: Polymerase ε
PTEN: Phosphatase and tensin homolog
RT-qPCR: Reverse Transcription Quantitative Real-Time Polymerase Chain Reaction
RNAPII: RNA polymerase II
TCGA: The Cancer Genome Atlas
TP53: Tumor protein p53
UCEC: Uterine corpus endometrial carcinoma
UDS: Unscheduled DNA synthesis
WCE: whole-cell extracts
XPC: Xeroderma Pigmentosum Complementation group C
XPE: Xeroderma Pigmentosum Complementation group E
