# Supplementary material for: PTEN-negative endometrial cancer cells protect their genome through enhanced DDB2 expression associated with augmented nucleotide excision repair
Source: BMC Cancer. 2023 May 4;23:399. doi: 10.1186/s12885-023-10892-5 (PMC10157935; doi:10.1186/s12885-023-10892-5)
Supplement: Supplementary file 2 — Supplementary Material 2 [file 12885_2023_10892_MOESM2_ESM.pdf]

Supplementary information

Title: PTEN-negative endometrial cancer cells protect their genome through enhanced DDB2 expression associated with augmented nucleotide excision repair.

Authors: Fathima Hameed J S[1,2], Anjali Devarajan[1], Devu Priya M S[1], Ahel Bhattacharyya[1], Mayur Balkrishna Shirude[2,3], Debasree Dutta[3], Parimal Karmakar[4], Ananda Mukherjee[1]

Address: [1]Rajiv Gandhi Centre for Biotechnology, Cancer Research Program, Thycaud PO, Poojappura, Thiruvananthapuram 695014, Kerala, India.

[2]Department of Biotechnology, Manipal Academy of Higher Education, Manipal, Karnataka State 576104, India.

[3]Rajiv Gandhi Centre for Biotechnology, Regenerative Biology Program, Thycaud PO, Poojappura, Thiruvananthapuram 695014, Kerala, India.

[4]Department of Life Science and Biotechnology, Jadavpur University, 188, Raja S.C. Mullick Road, Kolkata 700 032, West Bengal, India.

Address of correspondence:

Ananda Mukherjee

[1]Rajiv Gandhi Centre for Biotechnology, Cancer Research Program, Thycaud PO, Poojappura, Thiruvananthapuram 695014, Kerala, India.

Email: ananda@rgcb.res.in

Phone: +91 471 2529633

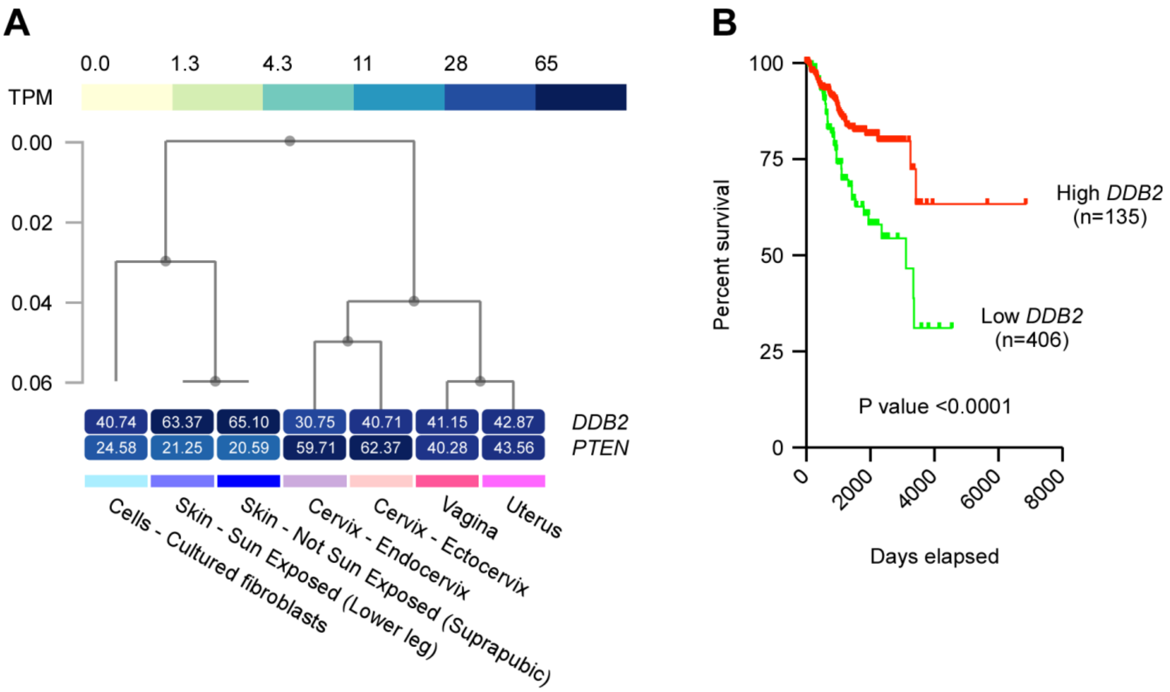

Supplementary Figure 1 related to Figure 1

Analysis of DDB2 expression in normal tissue and its correlation with EC patient survivability. A. The heat map was generated using the Genotype-Tissue Expression (GTEx) multi gene query function, showing mRNA expression values of DDB2 and PTEN in normal skin-related tissues and the female reproductive tract. The variable color scale represented mRNA expression values in TPM (transcript per kilobase million). The left axis represented the phylogenetic distance scale for the tissue lineages. B. Kaplan-Meier plots showing the correlation between mRNA expression of the DDB2 gene and EC patient survival. The survival outcomes of the two groups were compared by log-rank tests. Data were obtained from the pathology portal of the Human Protein Atlas database.

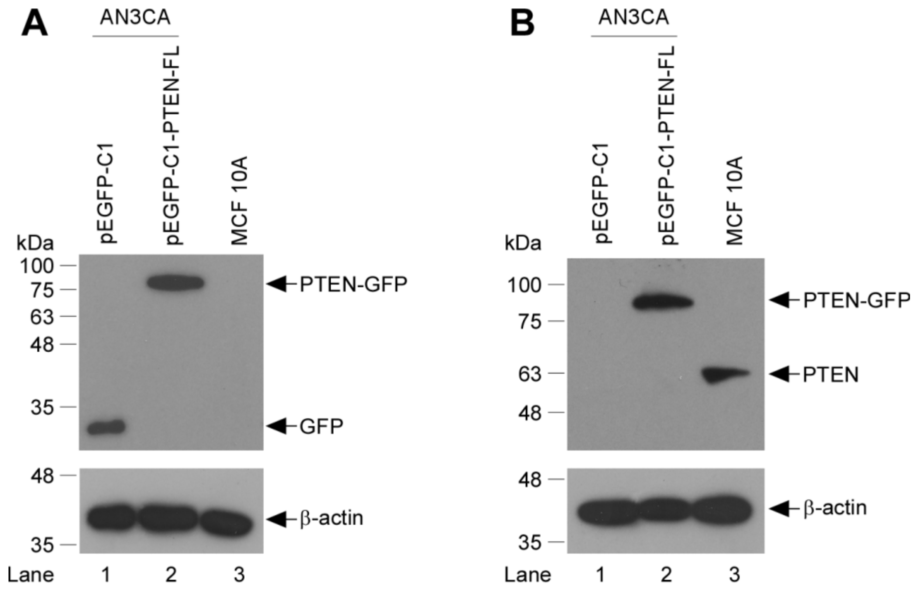

Supplementary Figure 2 related to Figure 2

Validation of fusion PTEN-GFP expression in AN3CA cells. A. Immunoblot probed with anti-GFP antibody in the AN3CA whole cell extract transiently overexpressing pEGFP-C1 (lane #1) and pEGFP-C1-PTEN-FL (lane #2) plasmids. MCF10A whole cell extract was used as a negative control for GFP expression (lane #3). β-actin was used as a loading control. B. Immunoblot probed with anti-PTEN antibody in the AN3CA whole cell extract transiently overexpressing pEGFP-C1 (lane #1) and pEGFP-C1-PTEN-FL (lane #2) plasmids. MCF10A whole cell extract was used as a positive control for endogenous PTEN expression (lane #3). β-actin was used as a loading control.

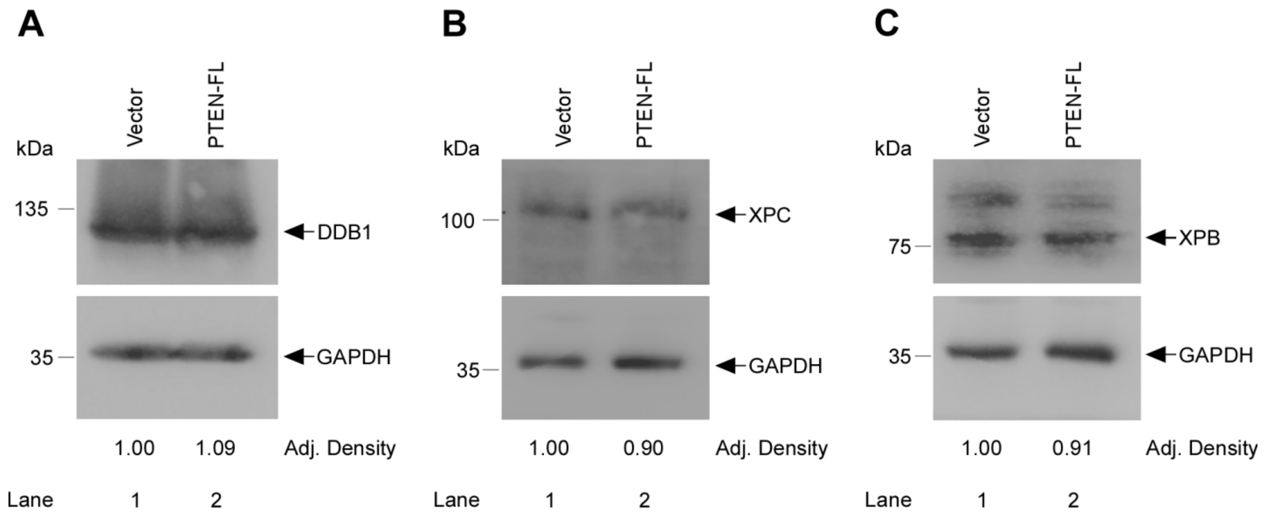

Supplementary Figure 3 related to Figure 2

Detection of GG-NER-associated proteins in the absence and presence of PTEN. A. Immunoblot probed with anti-DDB1 antibody in whole cell extracts of Vector and PTEN-FL cells. B. Immunoblot probed with anti-XPC antibody in whole cell extracts of Vector and PTEN-FL cells. C. Immunoblot probed with anti-XPB antibody in whole cell extracts of Vector and PTEN-FL cells. GAPDH was used as a loading control. The band intensities were measured as adjusted density (Adj. Density) relative to GAPDH.

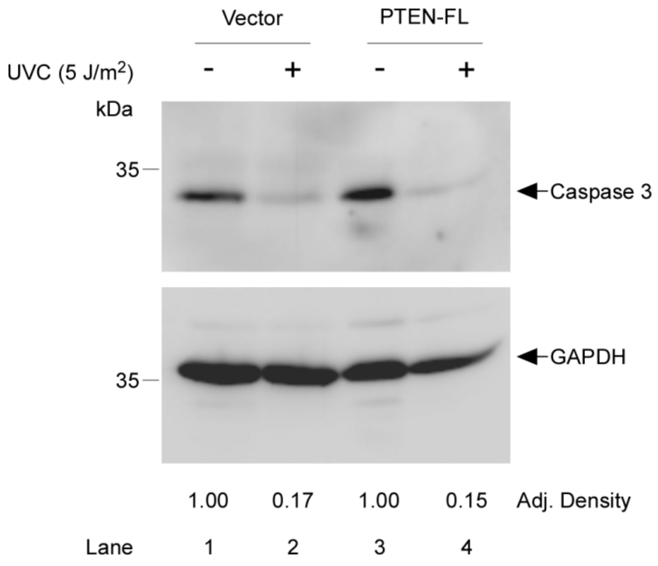

Supplementary Figure 4 related to Figure 3

UVC-induced apoptosis induction in the absence and presence of PTEN. Immunoblot probed with anti-Caspase 3 antibody in whole cell extracts of Vector and PTEN-FL cells detecting pro-caspase bands at 4 h post-UV irradiation. GAPDH was used as a loading control. The band intensities were measured as adjusted density (Adj. Density) relative to GAPDH.

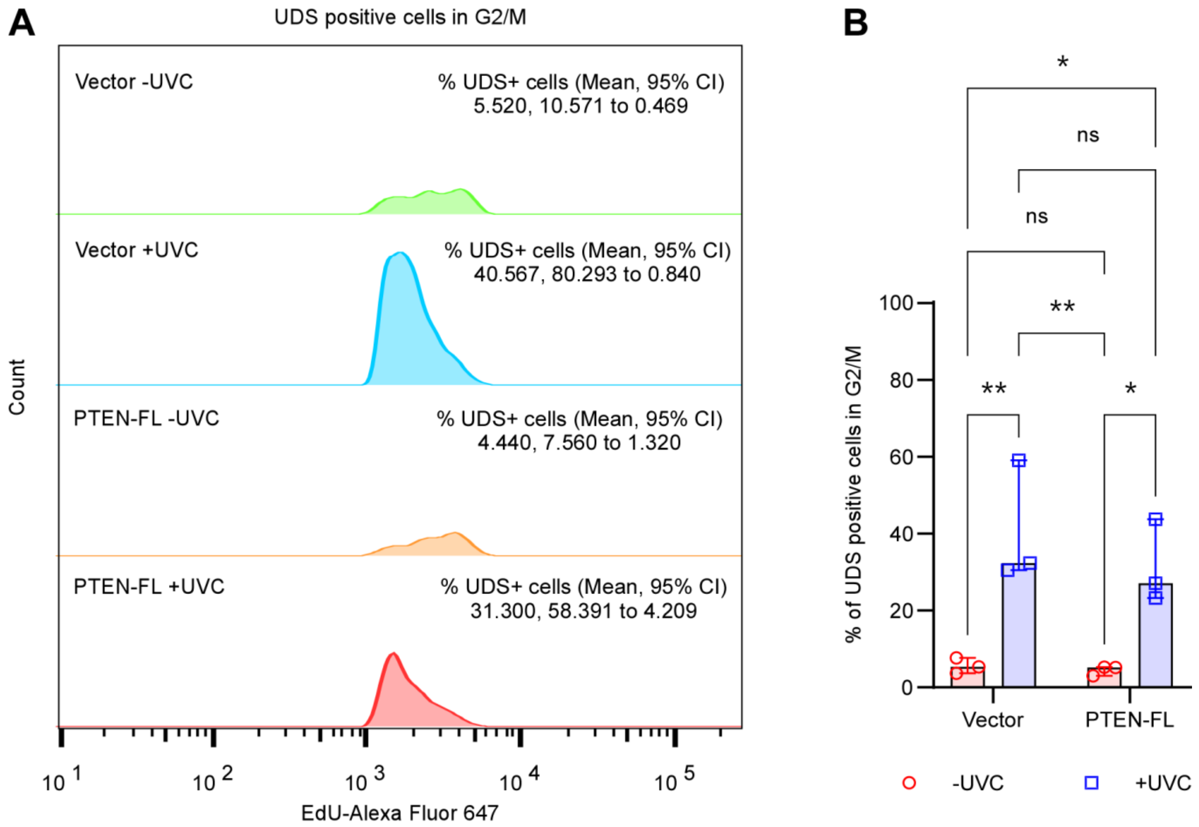

Supplementary Figure 5 related to Figure 4

Unscheduled DNA synthesis (UDS) in the G2/M phase of the cell cycle. A. Histograms represented the distribution of UDS-positive cells in the G2/M phase in each group, expressing the values as mean and 95% CI. B. The plot described the percentages of UDS-positive cells of three independent biological replicates as median and 95% CI. A two-way ANOVA test was performed to compare multiple groups. * P<0.05, ** P<0.01, ns, not significant.

PTEN uncropped

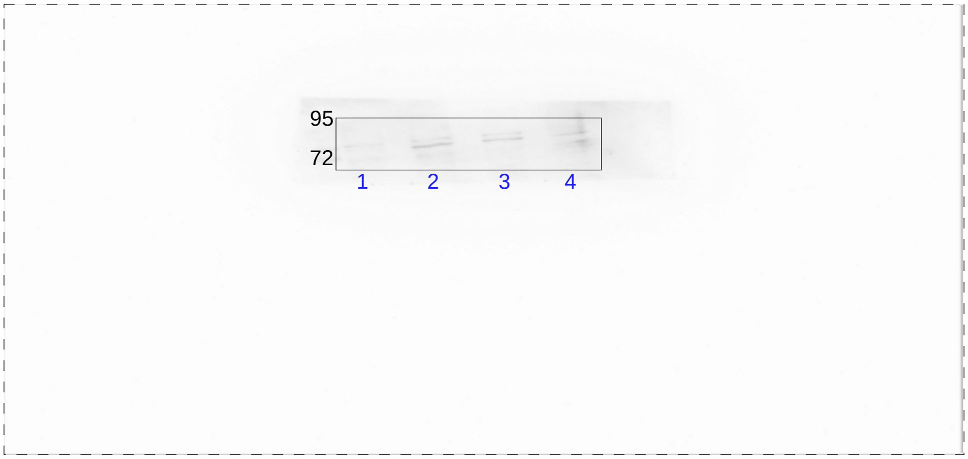

GAPDH uncropped

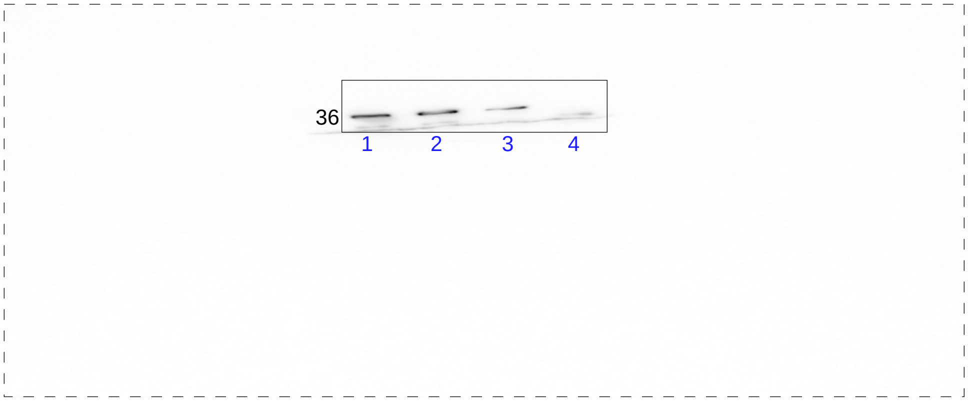

H3 uncropped

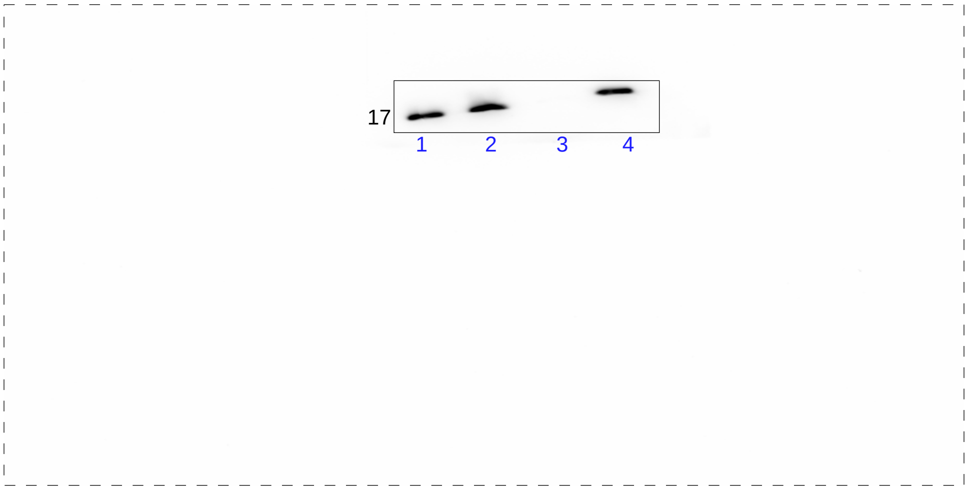

Figure 2B: Uncropped blots

pS473-Akt uncropped

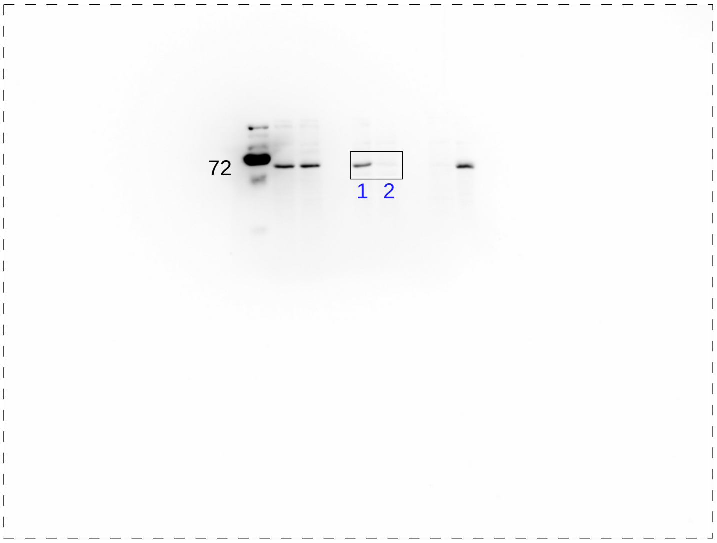

Total Akt uncropped

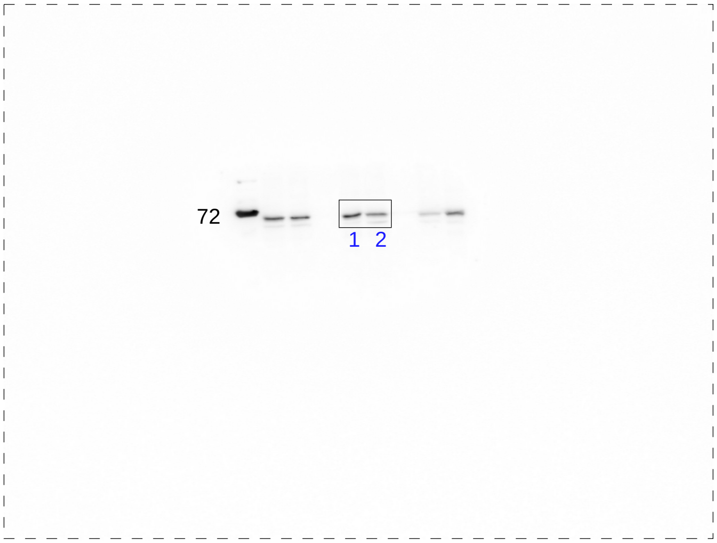

GAPDH uncropped

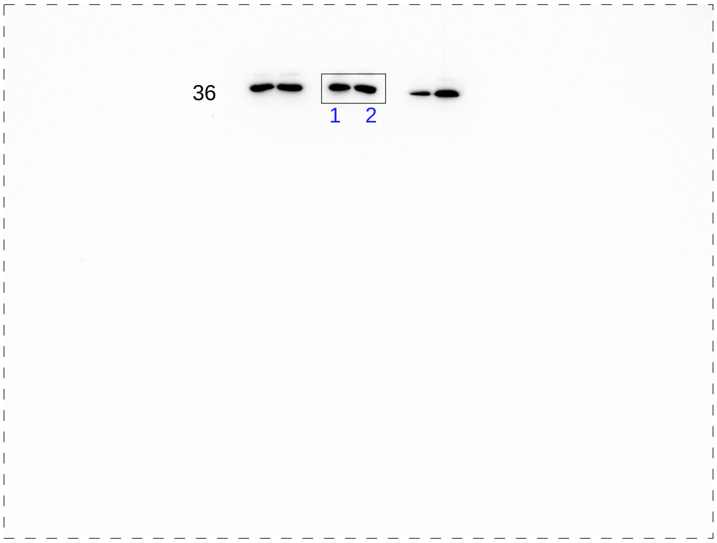

Figure 2C: Uncropped blots

DDB2 uncropped

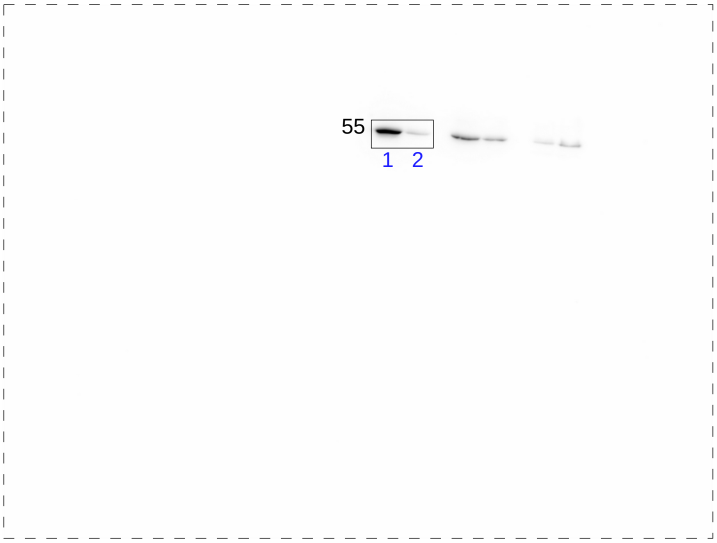

GAPDH uncropped

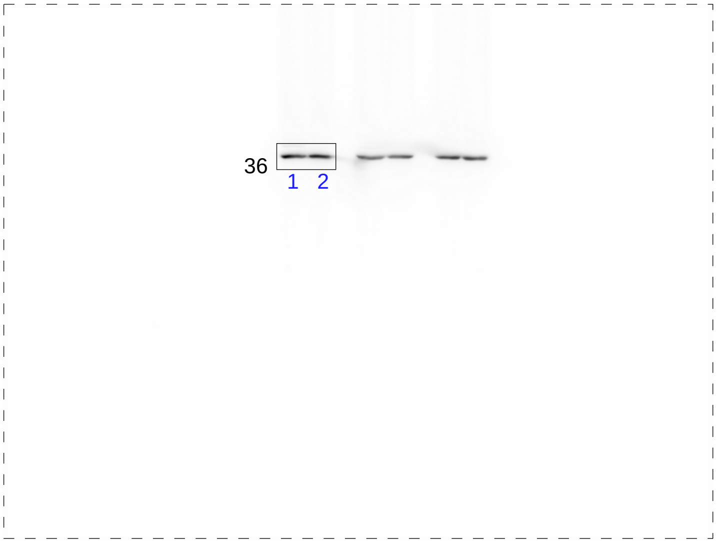

Figure 2E: Uncropped blots

DDB2 (Vector) uncropped

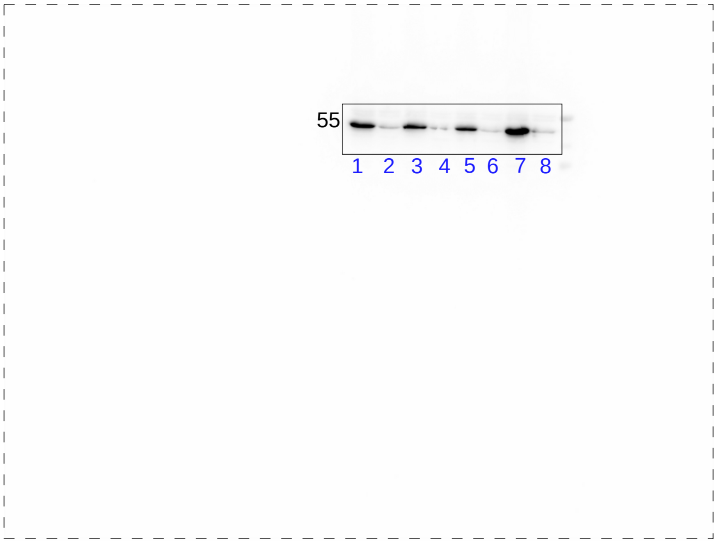

DDB2 (PTEN-FL) uncropped

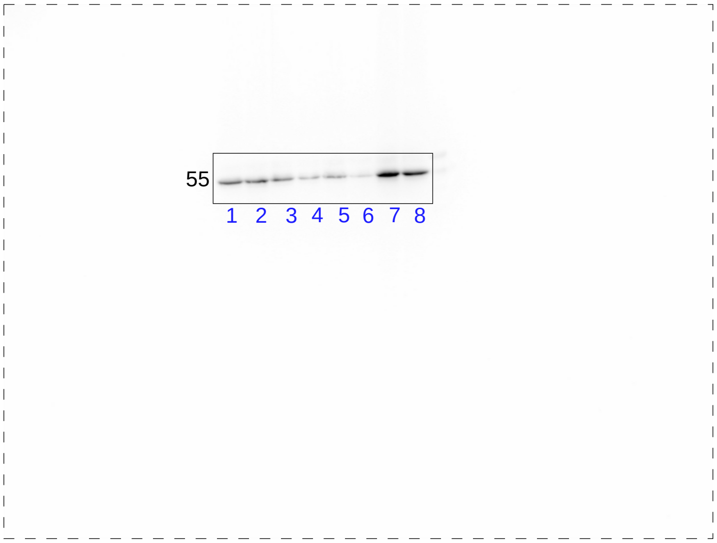

GAPDH (Vector) uncropped

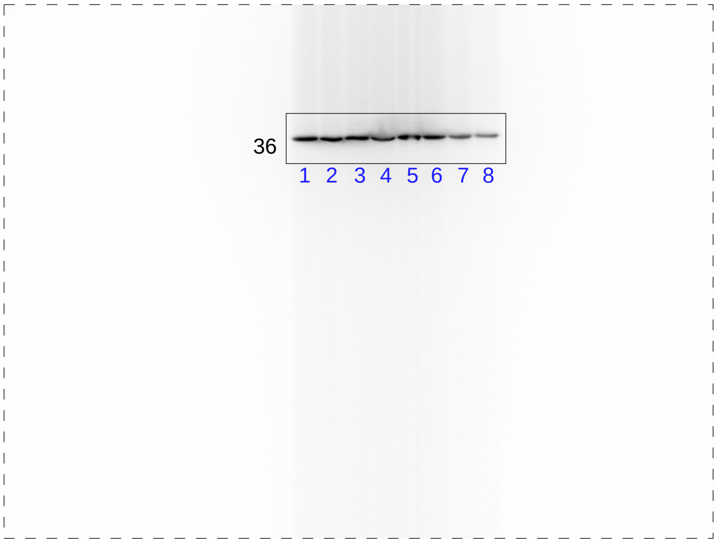

GAPDH (PTEN-FL) uncropped

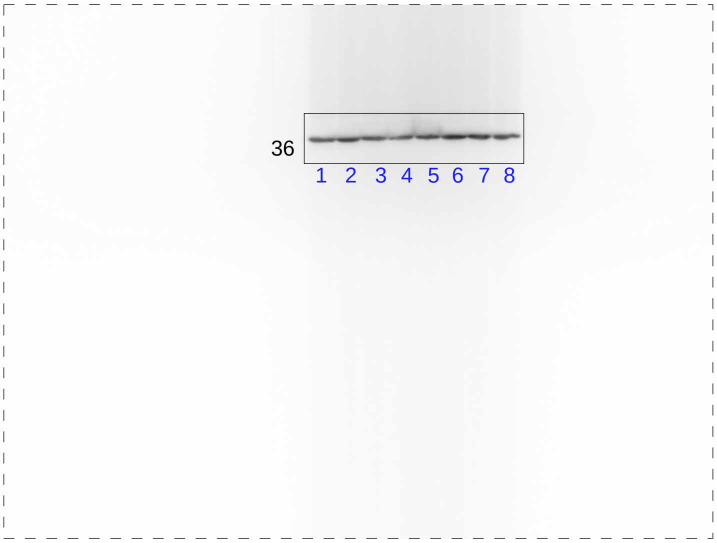

Figure 5C: Uncropped blots

DDB1 (Vector) uncropped

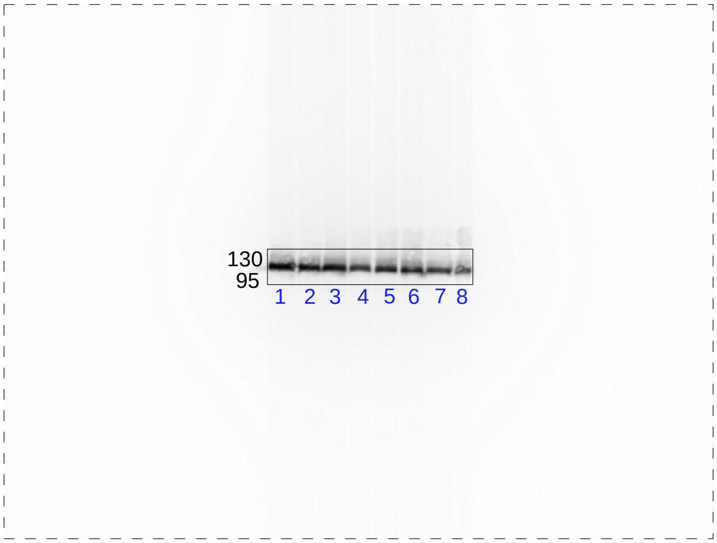

DDB1 (PTEN-FL) uncropped

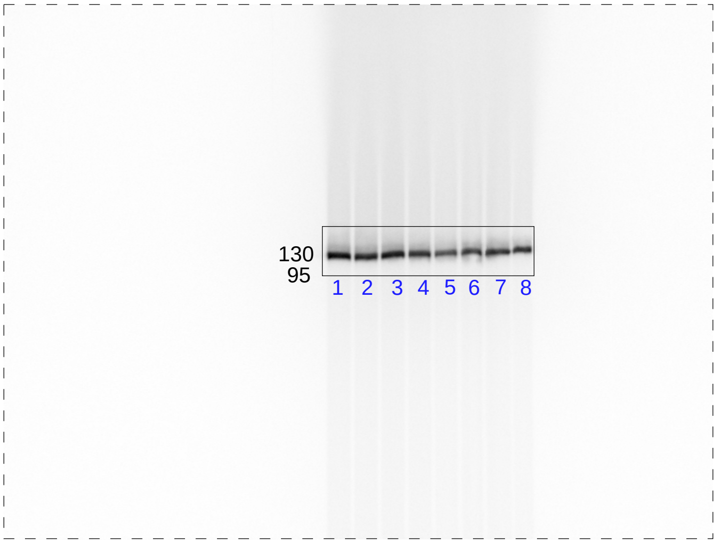

GAPDH (Vector) uncropped

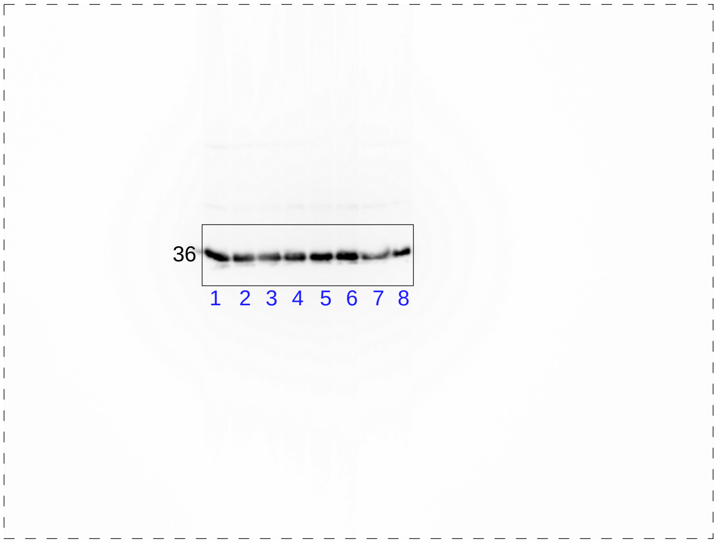

GAPDH (PTEN-FL) uncropped

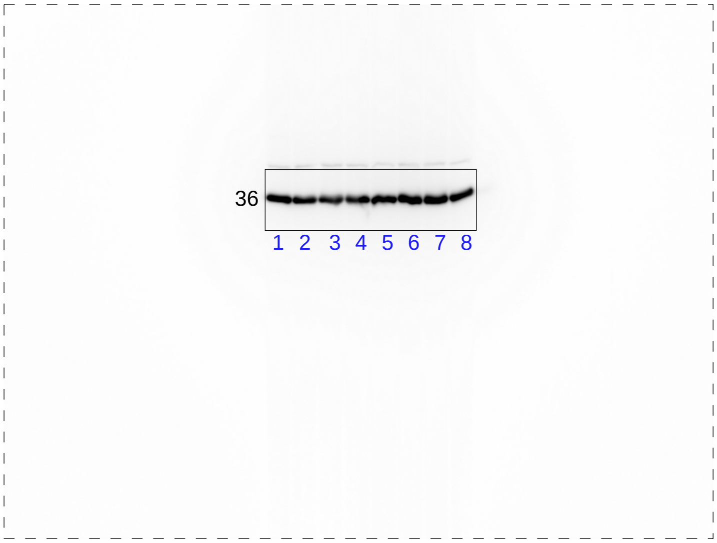

Figure 5E: Uncropped blots

GFP uncropped

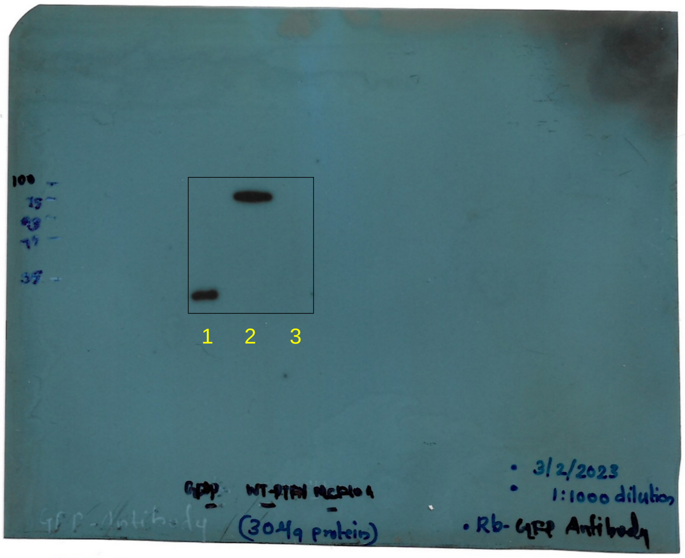

PTEN uncropped

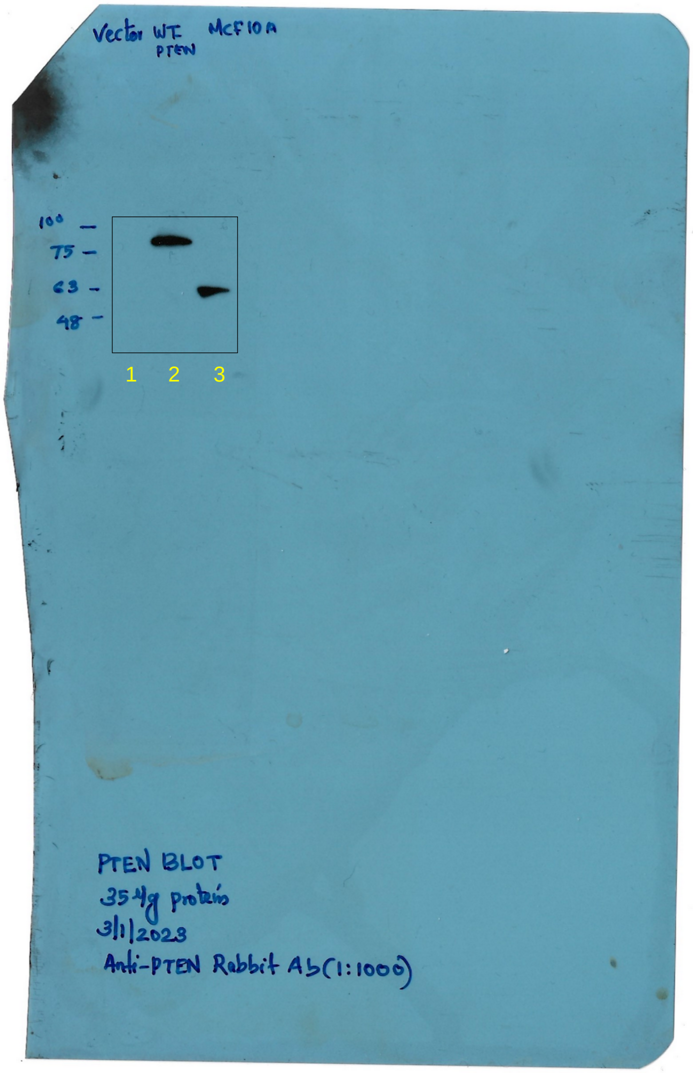

β-actin uncropped corresponding to GFP

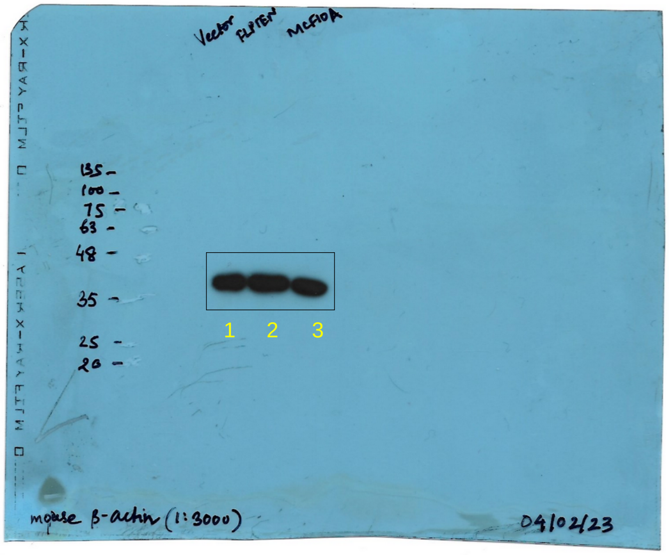

β-actin corresponding to PTEN

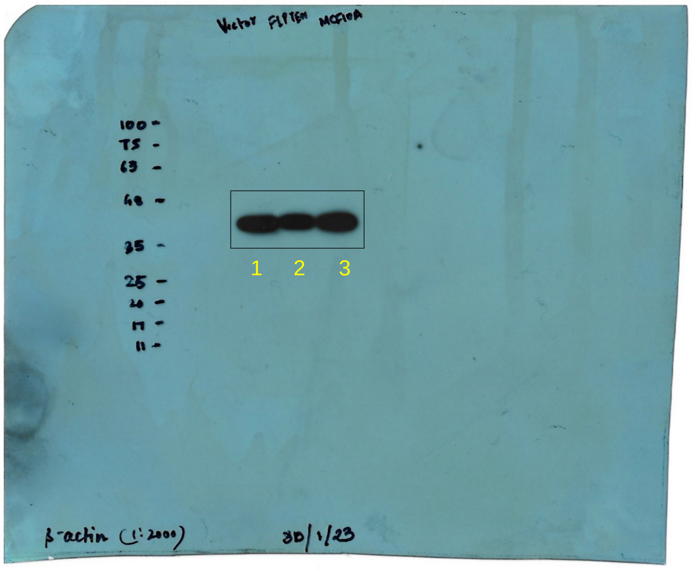

Supplementary Figure 2: Uncropped blots

DDB1 uncropped

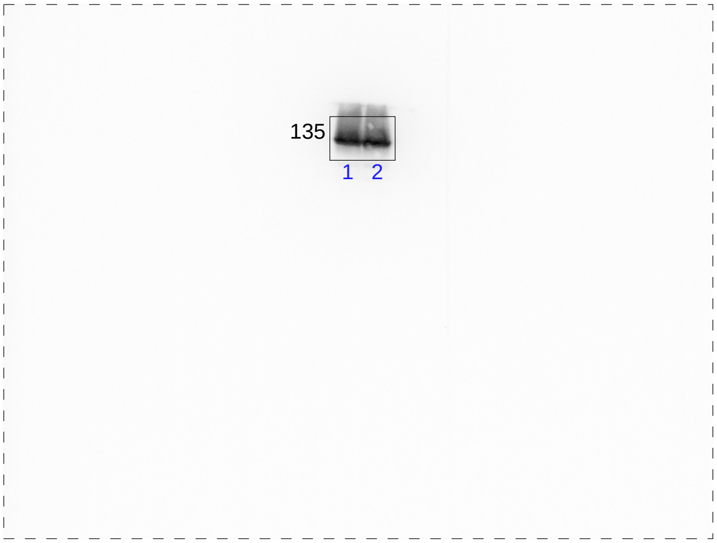

GAPDH uncropped corresponding to DDB1

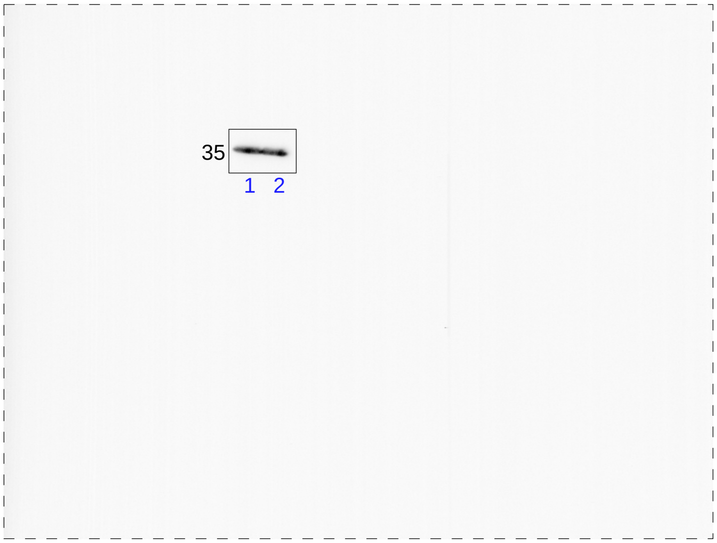

XPC uncropped

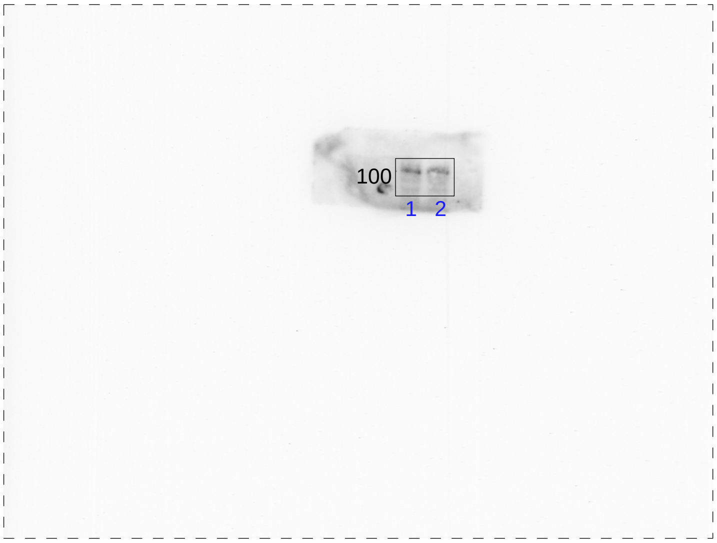

GAPDH uncropped corresponding to XPC

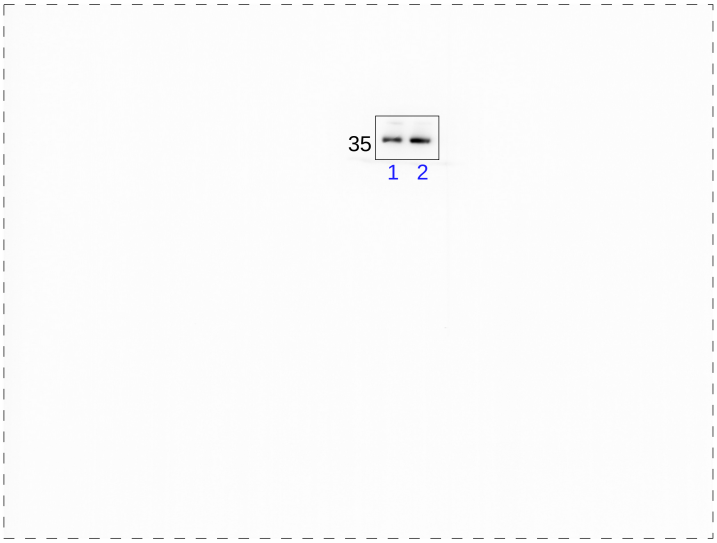

XPB uncropped

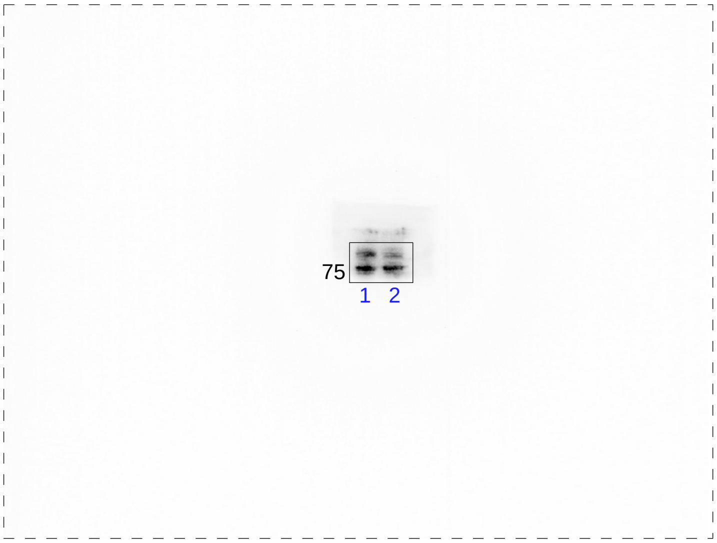

GAPDH uncropped corresponding to XPB

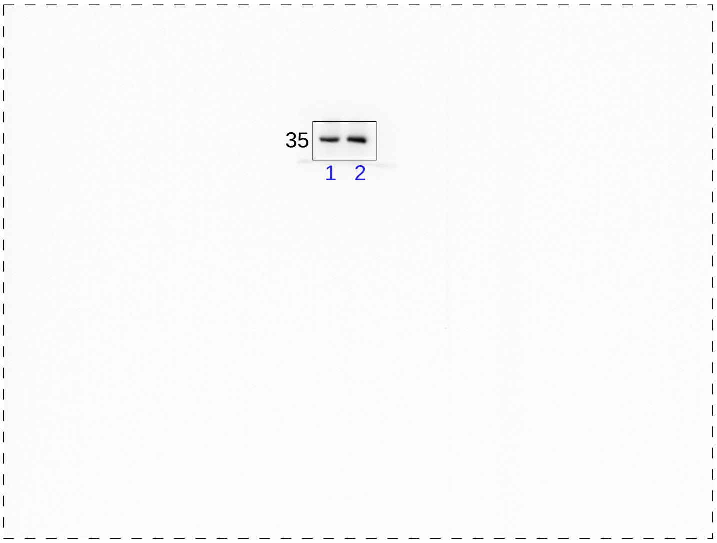

Supplementary Figure 3: Uncropped blots

Canpase-3 uncropped

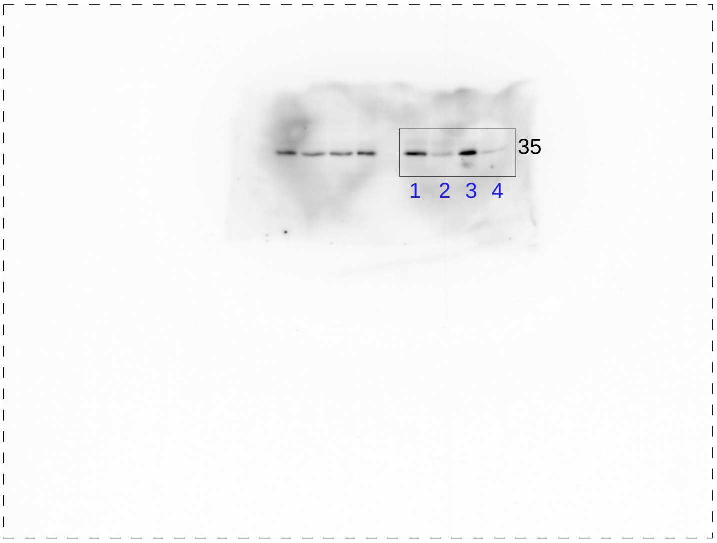

GAPDH uncropped corresponding to Caspase-3

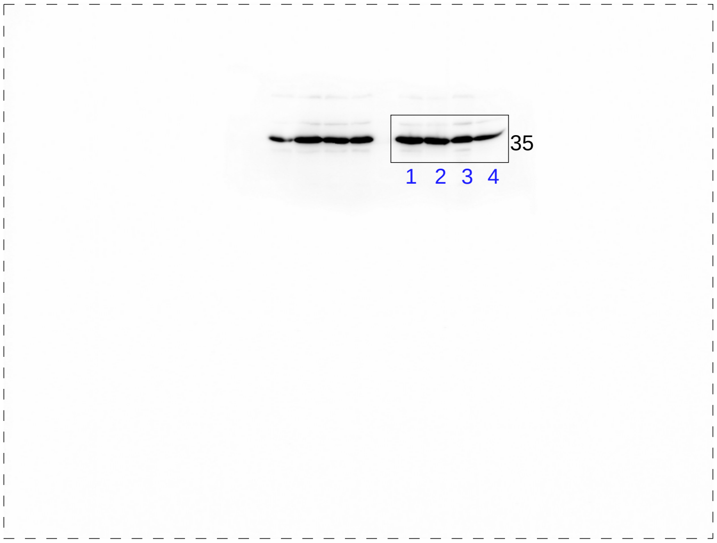

Supplementary Figure 4: Uncropped blots
